# Surface roughness modulates EGFR signaling and stemness of triple-negative breast cancer cells

**DOI:** 10.3389/fcell.2023.1124250

**Published:** 2023-03-08

**Authors:** Heizel Rosado-Galindo, Maribella Domenech

**Affiliations:** ^1^ Bioengineering Program, University of Puerto Rico-Mayagüez, Mayagüez, Puerto Rico; ^2^ Department of Chemical Engineering, University of Puerto Rico-Mayagüez, Mayagüez, Puerto Rico

**Keywords:** topography, EGFR signaling, TNBC, secretome, stemness

## Abstract

**Introduction:** Cancer stem cells (CSC), a major culprit of drug-resistant phenotypes and tumor relapse, represent less than 2 % of the bulk of TNBC cells, making them difficult to isolate, study, and thus, limiting our understanding of the pathogenesis of the disease. Current methods for CSC enrichment, such as 3D spheroid culture, genetic modification, and stem cell conditioning, are time consuming, expensive, and unsuitable for high-throughput assays. One way to address these limitations is to use topographical stimuli to enhance CSC populations in planar culture. Physical cues in the breast tumor microenvironment can influence cell behavior through changes in the mechanical properties of the extracellular matrix (ECM). In this study, we used topographical cues on polystyrene films to investigate their effect on the proteome and stemness of standard TNBC cell lines.

**Methods:** The topographical polystyrene-based array was generated using razor printing and polishing methods. Proteome data were analyzed and enriched bioprocesses were identified using R software. Stemness was assessed measuring CD44, CD24 and ALDH markers using flow cytometry, immunofluorescence, detection assays, and further validated with mammosphere assay. EGF/EGFR expression and activity was evaluated using enzyme-linked immunosorbent assay (ELISA), immunofluorescence and antibody membrane array. A dose-response assay was performed to further investigate the effect of surface topography on the sensitivity of cells to the EGFR inhibitor.

**Results:** Surface roughness enriched the CSC population and modulated epidermal growth factor receptor (EGFR) signaling activity in TNBC cells. Enhanced proliferation of MDA-MB-468 cells in roughness correlated with upregulation of the epidermal growth factor (EGF) ligand, which in turn corresponded with a 3-fold increase in the expression of EGFR and a 42% increase in its phosphorylation compared to standard smooth culture surfaces. The results also demonstrated that phenotypic changes associated with topographical (roughness) stimuli significantly decreased the drug sensitivity to the EGFR inhibitor gefitinib. In addition, the proportion of CD44+/CD24−/ALDH+ was enhanced on surface roughness in both MDA-MB-231 and MDA-MB-468 cell lines. We also demonstrated that YAP/TAZ activation decreased in a roughness-dependent manner, confirming the mechanosensing effect of the topographies on the oncogenic activity of the cells.

**Discussion:** Overall, this study demonstrates the potential of surface roughness as a culture strategy to influence oncogenic activity in TNBC cells and enrich CSC populations in planar cultures. Such a culture strategy may benefit high-throughput screening studies seeking to identify compounds with broader tumor efficacy.

## 1 Introduction

Triple-negative breast cancer (TNBC) is an aggressive type of breast cancer characterized by the absence of expression of progesterone receptor (PR), estrogen receptor (ER), and epidermal growth factor receptor-2 (HER2) (Yin et al., 2020). It affects approximately 20% of all breast cancer patients, and compared with other subtypes of breast cancer, it has a poorer prognosis, including worse overall survival and a higher relapse rate (Pareja et al., 2016; Wu et al., 2021). The lack of sensitivity to chemotherapy due to its molecular heterogeneity and rapid metastasis confers an aggressive phenotype and challenges effective therapeutic targets (Pareja et al., 2016; Yang et al., 2022). These unique attributes are mainly driven by gene mutations (e.g., p53, BRCA1), loss of expression of adhesion proteins (e.g., E-cadherin), and overexpression of epidermal growth factor receptor (EGFR) (Rakha et al., 2007; Li et al., 2019). Various studies have associated uncontrolled EGFR expression in TNBC with a poorer prognosis (Kallel et al., 2012; Park et al., 2014). The human EGFR family, comprising EGFR, HER2, ErbB3, and ErbB4, is responsible for stimulating cell proliferation, growth, differentiation, and survival (Gonzalez-Conchas et al., 2018). EGFR is overexpressed in approximately 50% of TNBCs, and when activated by the binding of epidermal growth factor (EGF) or other ligands, supports malignant growth, invasion, metastasis, and neovascularization (Weidner and Gasparini, 1994; Olayioye et al., 2000; Song et al., 2020). Thus, EGFR-targeted therapies have been developed to treat TNBC, yet disappointing results have been obtained in clinical trials (Duffy et al., 2012; von Minckwitz et al., 2005; Engebraaten et al., 2012; Crozier et al., 2016). A possible reason for this is that current high throughput drug screening assays are carried out on monolayer models, where cancer stem cells (CSCs) populations are as low as 2% (Boman and Wicha, 2008). Drug-resistant tumor cell populations, such as CSCs, are often underrepresented or excluded from therapy potency assays, limiting the broad assessment of drug efficacy and the identification of compounds that target these populations. Therefore, new approaches for enhancing CSCs and other resistant cell phenotypes are of vital importance in drug potency assays.

Currently, the gold standard method to obtain and enrich for CSCs *in vitro* is to cultivate tumor cells three-dimensionally (3D) as spheroids (Rosado-Galindo et al., 2021). Traditional methods for culturing 3D spheroids include hanging drops, rotary cell culture systems, and non-adherent surfaces (Mehta et al., 2012; Han et al., 2021). In the hanging drop method, cells are cultured as suspensions in droplets hanging upside down on the lid of a tissue culture plastic (TCP) plate (Foty, 2011), while spheroids cultured on non-adherent surfaces (e.g., agar-based (Gao et al., 2018), Poly-HEMA), and rotary bioreactors allow cells to aggregate because of the absence of cell-substrate interactions (Kopp et al., 2018; Bresciani et al., 2019). Many studies have demonstrated that 3D spheroid cultures provide a better representation of the tumor microenvironment (TME), which is reflected in enhanced drug resistance (comparable to PDX models) (Lanz et al., 2017; Brüningk et al., 2020), proliferation (Riedl et al., 2017; Pinto et al., 2020), and morphology (Kenny et al., 2007). However, spheroid culture can be challenging due to problems controlling spheroid size and uniformity, limited mass transfer, incompatibility with quantitative assays and generation techniques and complexity of the equipment used (Mehta et al., 2012; Han et al., 2021). Other methods, such as genetic modification (induced pluripotent cancer cells-iPCs), chemotherapy enrichment, growth of stem cells in cancer conditioned media, and hypoxia and reoxygenation cycles, have also been used to enrich CSCs populations (Azimi et al., 2017). However, research has demonstrated that reprogramming of cancer cells to CSCs can decrease their tumorigenic potency, and it is also costly and time consuming (Franco et al., 2016).

The use of physical cues to enrich CSCs and other aggressive tumor cell phenotypes could represent an alternative to overcome some of the challenges of standard culture methods used for CSCs. Extensive research has demonstrated that topographical cues of the microenvironment modulate gene expression (Wang et al., 2013; Radhakrishnan et al., 2015), proliferation (Sarwar et al., 2019), drug sensitivity (Sarwar et al., 2020), metastasis, and migration (Zhou et al., 2017) of cancer cells. Topographic patterns influence cytoskeletal deformation, adhesion, and migration. For example, Antmen *et al.* used breast cancer nuclear deformation on poly(methyl methacrylate) (PMMA) micropillars as a diagnostic tool to identify cell malignancy. MDA-MB-231 cells showed higher nuclear deformation compared to MCF7 cells and benign MCF10A cells, and the expression of adhesion genes changed inversely to the nuclear deformity (Antmen et al., 2019). Similarly, the migration speed of metastatic and non-metastatic breast cancer cells in micropatterns was used to study the invasive characteristics of the cells, where MDA-MB-231 cells displayed a higher migration speed in arc micropatterns compared to flat surfaces by inducing local asymmetry to the cells (Zhou et al., 2017). In addition, other studies have demonstrated that geometrical curvatures of cell-imprinted micropatterns increase the sensitivity of MCF-7 cells to doxorubicin (Shahriyari et al., 2020). In another study, anisotropic topographies such as gratings (grooves) induced enhanced proliferation of MCF-7 and MDA-MB-231 cells, but inhibited the proliferation of non-malignant cells *via* Rho-ROCK-myosin signaling, an effect that the authors called mechanically induced dormancy (Chaudhuri et al., 2016). Similarly, nano- and micro-grating patterns enhanced the proportion of the CD44+/CD24-subpopulation in MCF-7 cells (Tan et al., 2015) and decreased the expression of HER2 in HER2+ breast cancer cells BT-474 and SKBr3, validating the vast range of cell phenotypic changes induced by topographical stimuli (Daverey et al., 2022).

Although topographical cues are widely used to study cell behavior, their use has been hindered by several factors, including the biomaterials employed and the throughput. For example, substrates composed of natural materials (e.g., collagen and chitosan (Tudureanu et al., 2022)) or biodegradable polymers (e.g., hydroxyapatite and poly(ε-caprolactone) (Wei et al., 2020)) are less desirable because of cell remodeling, which causes the mechanical stimuli of the topographical cues in these materials to fade over time. Other biomaterials, such as PDMS and polyacrylamide, promote drug absorption and toxic bio-products that are undesirable for *in vitro* CSC cultures and drug assays (Caliari and Burdick, 2016; van Meer et al., 2017). Also, microfabrication methods employed to screen the effects of topographical cues are time consuming and require costly and specialized instrumentation, limiting their broad adoption into standard culture assays. In this study, we used razor printing and sanding methods to develop microtopographies in polystyrene (PS) films to stimulate phenotypic changes in TNBC cells. Polystyrene is the gold standard material used in cell biology. It is non-biodegradable, implying that mechanical stimulation will not fade over time, and is optically transparent, non-toxic, and inexpensive (Lerman et al., 2018). Our sticker-like PS micropatterns can be easily generalized across well-based culture platforms and employ user-friendly methods for fast prototyping (∼1.5 h) in a cost-effective manner. Here, we investigated the effect of surface microtopographies on the proteome and stemness phenotype of TNBC cell lines, MDA-MB-231 and MDA-MB-468. We demonstrated that topographical cues stimulated phenotypic changes in bulk tumor cells, including enhanced EGFR activity and enrichment of CSCs, supporting their potential for cell-based assays.

## 2 Materials and methods

### 2.1 Fabrication of polystyrene topographical array

The topographies evaluated in this study were the surface roughness, grooves, and curvature geometries generated on flat polystyrene (PS) films, as described in our previous work (Stallcop et al., 2018; Rosado-Galindo and Domenech, 2020). Briefly, a biaxially oriented, 0.19 mm thick PS film (ST311190/3, Goodfellow) and medical-grade tape (ARCare 90106) were used to produce the topographical surfaces. Grooves, zigzags, and spiral micropatterns were generated in the PS film using a cutting plotter (CE6000-40 Plus, Graphtec America, United States) equipped with a 0.9 mm diameter and 60° angle Graphtec blade (CB09UA). Surface roughness was generated on the PS films using an in-house fabricated press, with a sandpaper sheet attached to the top plaque. The PS film sheet was placed between the two plaques at a defined pressure and manually pulled out of the device. Topographical surfaces were plasma-treated using corona plasma treatment (Corona SB) to resemble the tissue culture plastic surface wettability. The depth of the razor-printed micropatterns and the surface roughness were measured and characterized using a Keyence 3D surface profiler (VK-X-1000, Keyence) and VK analyzer software (Keyence Corporation), respectively. The sticker-like substrates were taped to the bottom of the culture plates and sterilized using three cycles of 15 min exposure to UV light, followed by 1X phosphate buffer saline (PBS) wash step.

### 2.2 Cell culture

Triple-negative (ER-,PR-, HER-2-) breast cancer cell lines MDA-MB-231 and MDA-MB-468 were purchased from ATCC. Cells were expanded in DMEM high glucose (D6429, Sigma) supplemented with 10% heat-inactivated fetal bovine serum (FBS; F4135, Sigma) and 1% penicillin-streptomycin (P4333, Sigma), and maintained at 37°C and 5% CO2 in a humidified incubator. Cells were subcultured at 75%–80% confluency using 0.5% trypsin (59418C, Sigma). Cells were regularly tested for *mycoplasma* and verified to be mycoplasma-free using a MycoAlert *Mycoplasma* Detection Kit (LT07-318, Lonza).

### 2.3 Cell viability

The viability of TNBC cells was assessed using the Presto Blue Cell Viability Assay (A13261; Invitrogen). Cells were seeded at a density of 15,000 cells/cm2 on each topography in a 96-well culture plate, and their viability was measured after 5 days of culture in reduced serum media (2% FBS) following the manufacturer’s protocol. Briefly, half of the culture medium was replaced with fresh media containing the presto blue reagent (1:10), and the cells were incubated at 37 °C for 2 h. Finally, the fluorescence intensity of the collected samples was measured using a Spark multiplate reader. Fluorescence intensity was normalized to the total number of cells per well.

### 2.4 Cell morphology

Cell morphology was analyzed by fluorescence staining of the cytoskeleton. Cells were seeded at a density of 9,000 cells/cm2 on each substrate bound to the bottom of a well of a 96-well plate and allowed to attach overnight. The next day, the culture medium was changed to a reduced-serum formulation containing 2% FBS, and the cells were cultured for 5 days. Cells were then fixed for 15 min in 4% paraformaldehyde (sc-281692, Santa Cruz Biotechnology) and permeabilized using 0.2% Triton X-100 (T8787, Sigma) in PBS for an additional 15 min at room temperature. Cytoskeleton staining was performed by incubating the ActinRed™ 555 ReadyProbes reagent (R37112, Invitrogen) for 30 min, and cell nuclei were counterstained with Hoechst 33342 (1:1000 dilution; H1399, Invitrogen) for 10 min at room temperature. Fluorescent images of the cells were acquired at 20X images using a Keyence BZ-X800 fluorescence microscope. Cell morphology (cell elongation and area) were measured using CellProfiler software (Version 4.0.6).

### 2.5 EdU proliferation assay

Cell proliferation was measured using a Click-itTM Plus EdU Cell Proliferation Kit for Imaging (C10639, Thermo Fisher). The cells were seeded at a density of 15,000 cells/cm2 on each topography of a 96-well culture plate and allowed to attach overnight. The next day, the culture medium was changed to a reduced-serum formulation containing 2% FBS, cells were cultured for 5 days, and EdU staining was performed using the protocol described by the manufacturer. At first glance, the cells were incubated for 2 h with 10 µM EdU working solution. Cells were then fixed for 15 min in 4% paraformaldehyde (sc-281692, Santa Cruz Biotechnology) and permeabilized using 0.2% Triton X-100 (T8787, Sigma) in PBS for an additional 15 min at room temperature. Cells were washed twice with 3% BSA (A9647,Sigma) in PBS solution, and the Click-it^®^ Plus reaction cocktail was added and incubated for 30 min at room temperature in the dark. Finally, the reaction cocktail was washed once with 3% BSA in PBS, and cell nuclei were counterstained with Hoechst 33342 (H1399, Invitrogen) at a 1:1000 dilution for 10 min at room temperature. Fluorescent images were obtained using a Keyence BZ-X800 microscope and analyzed using the ImageJ software (Version 1.53a).

### 2.6 Collection of conditioned media and lysate for cytokine analysis

Cells were seeded on each substrate bound to the bottom well of a 12-well plate at a density of 100,000 cells/well and allowed to attach overnight. The culture medium was changed to a reduced-serum formulation containing 2% FBS, and the cells were cultured for 5 days with media replenishment on day 3. Afterwards, conditioned media were collected, cells were lysed, and samples were prepared and sent to RayBiotech Life, Inc. for quantification of 200 human proteins (QAH-CAA-4000-1, RayBiotech Life, Inc.,). Initial cutoff of differentially expressed proteins was performed by selecting the proteins with fold change >2 for overexpressed proteins or <0.5 for downregulated proteins and adjusted *p*-values of 0.05 for edgeR analysis using R software (R 4.2.1). Enriched pathways and biological significance were analyzed using the Gene Ontology tool enrichGO from the clusterprofiler Ver 3.15 package from Bioconductor (R software), and proteins were deemed significant if the *p*-value was <0.01.

### 2.7 ALDH Detection Assay

The expression of aldehyde dehydrogenase (ALDH) was measured using the AldeRed ALDH Detection Assay (SCR150,Millipore) following the manufacturer’s protocol. TNBC cells were seeded on topographical surfaces attached to 24-well plates at a density of 52,000 cells/well and allowed to attach overnight. The culture medium was then changed to a reduced-serum formulation containing 2% FBS, and cells were cultured for 5 days. Briefly, the cells were washed once with PBS and detached from each well plate using 0.5% trypsin EDTA. The cells were then resuspended in cell culture media (10% FBS) and the cell number was adjusted to 2 × 10^5^ cells per sample. Cells were centrifuged and resuspended in AldeRed assay buffer, and the AldeRed substrate was added to each sample. The samples were incubated at 37°C for 45 min, resuspended in ice-cold AldeRed assay buffer, and maintained on ice during analysis. The cells were analyzed using flow cytometry (BD Accuri™ C6 Plus). Diethylaminobenzaldehyde (DEAB), an ALDH inhibitor, was used as a negative control to gate the ALDH + population.

### 2.8 Immunofluorescent staining

*ALDH staining:* Immunofluorescence staining of TNBC cells cultured on topographical surfaces was performed at room temperature. Cells were washed with PBS and fixed for 15 min in 4% paraformaldehyde, followed by permeabilization with 0.2% Triton X-100 (T8787, Sigma) in PBS for an additional 10 min. The cells were resuspended in blocking buffer 3% BSA in PBS + 0.1% Tween20 (P9416, Sigma) and incubated for 1 h. For staining, the cells were incubated with anti-ALDH (sc-166362, Santa Cruz Biotechnology, Inc.) at a ratio of 1:250 in 3% BSA in PBS + 0.1% Tween20 (P9416, Sigma) solution for 1 h at RT. The cells were then incubated with anti-mouse Alexa Fluor 647 secondary antibody (ab150115, Abcam) at a ratio of 1:500 in 3% BSA in PBS + 0.1% Tween20 solution for 1 h. Lastly, cells were counterstained with Hoechst 33342 (1:1000 dilution) and washed three times with PBS. Fluorescent images were acquired at 10X magnification using a Keyence BZ-X800 fluorescence microscope. Fluorescent intensity was measured using ImageJ software (Version 1.53a).

*EGFR staining:* Cells were washed with PBS and fixed for 15 min in 4% paraformaldehyde, followed by permeabilization with 0.2% Triton X-100 (T8787, Sigma-Aldrich) in PBS for an additional 10 min. The cells were resuspended in blocking buffer 3% BSA in PBS + 0.1% Tween20 (P9416, Sigma) and incubated for 1 h. For staining, the cells were incubated with anti-EGFR (528) (sc-120, Santa Cruz Biotechnology, Inc.) at a ratio of 1:250 in 3% BSA in PBS + 0.1% Tween20 (P9416, Sigma) solution overnight at 4°C. The cells were then incubated with anti-mouse Alexa Fluor 647 secondary antibody (ab150115, Abcam) in 3% BSA in PBS + 0.1% Tween20 solution for 1 h. Lastly, cells were counterstained with Hoechst 33342 (1:1000 dilution) and washed three times with PBS. Fluorescent images were acquired at 20X images using a Keyence BZ-X800 fluorescence microscope. The fluorescence intensity was measured using ImageJ software (version 1.53a).

*YAP/TAZ Staining:* Cells were washed with PBS and fixed for 15 min in 4% paraformaldehyde, followed by permeabilization with 0.5% Triton X-100 (T8787, Sigma) in PBS for an additional 10 min. The cells were resuspended in blocking buffer 3% BSA in PBS + 0.1% Tween20 (P9416, Sigma) and incubated for 1 h. For staining, the cells were incubated with anti-YAP/TAZ (D24E4, Cell Signaling) at a ratio of 1:250 in 3% BSA in PBS + 0.1% Tween20 (P9416, Sigma) solution for 1 h at RT. The cells were then incubated with anti-rabbit Alexa Fluor 488 secondary antibody (ab150077, Abcam) at a ratio of 1:500 in 3% BSA in PBS + 0.1% Tween20 solution for 1 h. Lastly, cells were counterstained with Hoechst 33342 (1:1000 dilution) and washed three times with PBS. Fluorescent images were acquired at 10X magnification using a Keyence BZ-X800 fluorescence microscope. Fluorescent intensity was measured using ImageJ software (Version 1.53a).

### 2.9 Mammosphere assay

The mammosphere assay was performed as previously described by Shaw et al. al (Shaw et al., 2012). First, TNBC cells were cultured on flat smooth surfaces (TCP) and surfaces with roughness Ra = 1.5 µm for 5 days in DMEM supplemented with 2% FBS and 1% P/S. For the first generation of mammospheres, 10,000 tumor cells were seeded in ultra-low attachment (ULA) well plates and cultured in 2 mL of mammosphere media (MEBM media (CC-3153, Lonza) supplemented with 0.02 ug/mL of bFGF (100-18B, Peprotech), 0.001 mg/mL of hydrocortisone (H6909, Sigma-Aldrich), 0.005 mg/mL recombinant human insulin (I9278, Sigma-Aldrich), 0.02ug/mL EGF (E4127, Sigma-Aldrich), 1X B27 supplement minus vitamin A (12587-010, Gibco), and 0.005 mg/mL Gentamicin (15750-060, Gibco) and incubated at 37°C/5% CO2 for 7 days. Afterwards, the cells were disaggregated using 0.5% trypsin + 0.2% EDTA and filtered through a 5 mL polystyrene round-bottom tube with a cell strainer cap for single-cell sorting. For the second-generation passage, 4,000 cells per well were seeded in ULA 24-well plates using 1 mL media and cultured using mammosphere media treated with vehicle (DMSO) and Docetaxel 10 µM (4056, R&D Systems) for seven additional days. Images of mammospheres were taken at 10X and 20X magnification using a Keyence BZ-X800 microscope. Whole-well analysis was carried out to quantify the total number of mammospheres >50 µm in diameter using the particle analyzer of ImageJ software (Version 1.53a).

### 2.10 EGF/EGFR ELISAs

EGF and EGFR concentrations in the conditioned media and cell lysates of MDA-MB-231 and MDA-MB-468 cells were measured using enzyme-linked immunosorbent assay (ELISA) according to the manufacturer’s instructions (ELH-EGF-1/ELH-EGFR-1, RayBiotech Life, Inc.,). Briefly, cells were seeded on topographical surfaces attached to 96-well plates at a density of 7,000 cells/well and allowed to attach overnight. The culture medium was changed to DMEM supplemented with 2% FBS and 1% P/S and incubated for 5 days with medium replenishment on day 3. Afterwards, the conditioned media were collected and cells were lysed using a solution of 1X RIPA buffer (R0278, Sigma) and protease inhibitor cocktail (K271, BioVision). Samples were centrifuged for 10 min at 10,000 rpm and 4°C, and the supernatant was collected and diluted 3–5-fold with the provided dilution buffer in the corresponding EGF/EGFR-coated wells. First, the samples were incubated for 2.5 h at room temperature and then washed 3 times with the provided wash buffer. Next, a biotinylated anti-human EGF/EGFR antibody was added and incubated for another hour, and an additional washing step was performed. Afterward, HRP-conjugated streptavidin was added and incubated for 45 min, followed by another washing step. Finally, a TBM substrate was added and incubated for 30 min, the stop solution was added, and the absorbance was measured at 450 nm using a Tecan Spark multi-plate reader. EGF and EGFR concentrations were adjusted to the total protein concentration of each sample. The total sample concentration was measured using a Pierce BCA Protein Assay Kit (23335, Fisher), following the manufacturer’s protocol.

### 2.11 Flow cytometry

CD44/CD24 and EGFR expression were measured in TNBC cells cultured on topographical substrates using flow cytometry. Cells were harvested and fixed for 15 min in 4% paraformaldehyde. Then cells were washed with PBS and centrifuged for 5 min at 2000 rpm. The supernatant was removed and 500ul of 0.1% Triton X-100 0.5% BSA-PBS was added to the cell pellet and incubated for 10 min at RT. Afterwards, cells were washed with 0.5% BSA-PBS and cells were spun down at 2,500 rpm for 5 min. Cell pellet was resuspended in 100ul/tube of (1:250) anti-HCAM Fluor^®^ 488 (sc-7297 AF488, Santa Cruz Biotechnology, Inc.) and (1:250) Anti-CD24 Fluor^®^ 594 (sc-19585 AF594, Santa Cruz Biotechnology, Inc.) or (1:100) anti-EGFR (528) (sc-120, Santa Cruz Biotechnology, Inc.) diluted in 0.5% BSA-PBS solution overnight at 4°C. Cells were washed with 1 mL of 0.5% BSA-PBS solution and centrifuged for 5 min at 2,500 rpm. CD44/CD24 stained samples were resuspended in 100ul/tube of PBS. EGFR stained samples were incubated with (1:250) anti-mouse Alexa Fluor 488 secondary antibody diluted in 0.5% BSA-PBS solution for 1 h at RT. Cells were finally washed with 1 mL of 0.5% BSA-PBS solution and centrifuged for 5 min at 2,500 rpm and resuspended in 100ul/tube of PBS. Isotype controls were incubated with corresponding secondary antibodies only. The samples were analyzed using flow cytometry (BD Accuri™ C6 Plus).

### 2.12 EGFR phosphorylation array

EGFR Phosphorylation patterns of TNBC cells cultured on topographical substrates were measured using the Human EGFR Phosphorylation Antibody Array Membrane (Ab134005, Abcam), following the manufacturer’s protocol. TNBC cells were seeded on topographical surfaces and cultured in a reduced-serum formulation containing 2% FBS for 5 days. Cells were then lysed using a solution of 1X RIPA buffer (R0278, Sigma) and protease inhibitor cocktail (K271, BioVision). The total sample concentration was measured using a Pierce BCA Protein Assay Kit (23335, Fisher) and adjusted to 200 ug/mL per sample. Membranes were blocked for 1 h at room temperature and gentle shaking with the assay blocking buffer. The samples were then incubated overnight at 4°C and washed with assay buffer. Biotin-conjugated anti-EGFR was then added to each membrane and incubated for 2 h at room temperature, followed by washing. HRP-conjugated streptavidin was added to the membranes and incubated for an additional 2 h at room temperature, followed by washing. Finally, the membranes were incubated for 2 min and gently shaken with the detection buffer, and chemiluminescence signals were detected using the ChemiDoc XRS + imaging system (BioRad).

### 2.13 Drug inhibition study

A dose-response assay was performed to investigate the effect of surface topography on the sensitivity of cells to the EGFR inhibitor gefitinib (3000/10, Tocris Bioscience). Cells were seeded at a density of 15,000 cells/cm2 on each culture surface in a 96-well culture plate and allowed to attach overnight. The next day, the culture medium was changed to DMEM supplemented with 2% FBS, 1% P/S, and the following drug concentrations:0 µM, 0.1 µM, 1 µM, 5 µM, 10 µM, 20 µM, 50 µM, and 100 µM. After 5 days of incubation, cell viability was assessed using the Presto Blue™ cell viability assay (A13261, Invitrogen).

### 2.14 Statistics

Statistical analysis was performed using Graph Pad Prism 9.0 (GraphPad Software Inc., San Diego, United States) and the statistical software R version 4.2.0. The results are presented as the mean ± standard error of the mean (s.e.m), and differences between groups were analyzed using the Mann-Whitney non-parametric *t*-test with a significance level of ɑ = 0.05.

## 3 Results

### 3.1 Topographical surfaces alter the proliferation of TNBC cells

**FIGURE 1:**
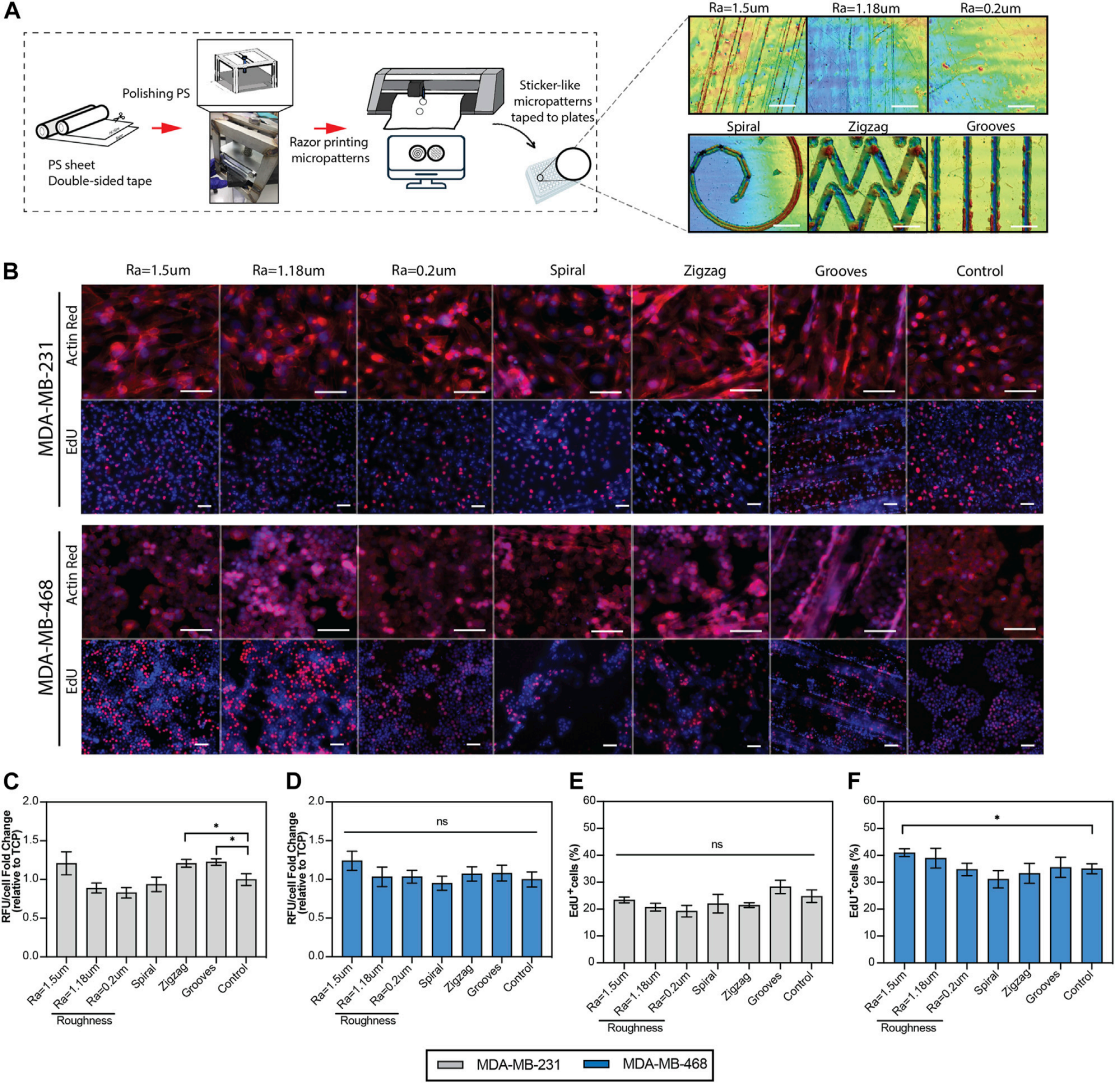
Viability and proliferation of the TNBC cells cultured on the topographical substrates. **(A)** Diagram of the generation of the polystyrene topographical substrates using razor-printing and polishing methods and their characterization by laser confocal scanning 3D microscopy. **(B)** Top: Representative immunofluorescence images of TNBC cells cultured on the topographical substrates and TCP (control). The cytoskeleton of the cells was stained with actin red (red) and the nuclei with hoechst (blue). Scale bar 100 µm. Bottom: Representative immunofluorescence images of the proliferation of TNBC cells, where hoechst (blue) represents all the cells and EdU (red) newly synthesized DNA. Scale bar = 50 µm. **(C, D)** PrestoBlue viability assay of TNBC cell lines, quantified as relative fluorescence units per cell, relative to the control (TCP). **(E, F)** Quantification of the proliferation of TNBC cells, depicted by the percentage of EdU positive cells. TNBC cells were cultured for 5 days in reduced serum (2% FBS) on the topographical substrates and TCP (control). Error bars depict the mean ± SEM of 2-3 independent experiments with n = 3-4 samples. *T*-test with Mann-Whitney non-parametric test. Asterisk (*) represent *p*-values < 0.05, (**) *p*-values < 0.01, (***) *p*-values < 0.001 and (****) *p*-values < 0.0001.

To investigate the effect of surface microtopography on cell behavior, we first fabricated PS sticker-like topographies with three different levels of roughness generated by microscopic linear scratches characterized by their average surface roughness (Ra). In addition, geometrical micropatterns (depicted by spiral and zigzags) and grooves were generated on the PS using razor printing and polishing methods (Figure 1A), as described previously (Rosado-Galindo and Domenech, 2020). Human TNBC cell lines, MDA-MB-231 and MDA-MB-468, were cultured on topographical surfaces, and viability, morphology, and proliferation were measured as initial indicators of potential phenotypic changes in the cells. The results showed that the spread of both cell lines on the topographical surfaces was comparable to their counterparts on the standard TCP surface (control), as depicted by cytoskeleton staining (actin red) in Figure 1B. Morphological features of the cells (e.g., cell area and elongation) were not significantly affected by the topographical surfaces, except for MDA-MB-231 cells cultured on substrates with an average roughness of 1.5 µm and grooves where the area was significantly increased and cell elongation was slightly higher than that of TCP (Supplementary Figure S1). Enhanced elongation of MDA-MB-231 cells was expected, as this morphological feature correlates well with their intrinsic invasive phenotype. The viability of TNBC cells was equivalent to that of the control across the topographical surfaces, except for a significant increase of 20% in MDA-MB-231 cells cultured in grooves and zigzag micropatterns (Figure 1C). Both cell lines showed an average 15% increase in cell growth on surfaces with Ra = 1.5 µm but the data were deemed significant only for MDA-MB-468 cells (Figure 1D). In agreement with this finding, the number of MDA-MB-468 cells in the growth S-phase (depicted by the percentage of EdU + cells) were significantly increased for a surface roughness of Ra = 1.5 µm (Figure 1B-EdU panel and Figure 1F). This pattern was also observed in the rest of the topographical surfaces, where proliferation increased or decreased in accordance with an increase or decrease in viability (Figures 1E, F), indicating that topographical cues can influence cell growth rates. Since not all the cells are exposed to the topographical stimuli in grooves and curved geometries, cell proliferation analysis was further stratified by regions within the geometrical micropatterns (e.g., straight lines vs. corners, degree of curvature, or outer vs. inner areas). However, no significant differences in cell proliferation were observed within regions (Supplementary Figure S4A), suggesting that changes in cell proliferation of the bulk cell population are likely driven by secreted factors from mechano-stimulated cells that diffuse within the culture media impacting neighbor cells.

### 3.2 Surface roughness enhances bioprocesses associated with EGF/EGFR signaling, stemness and inflammation

**FIGURE 2:**
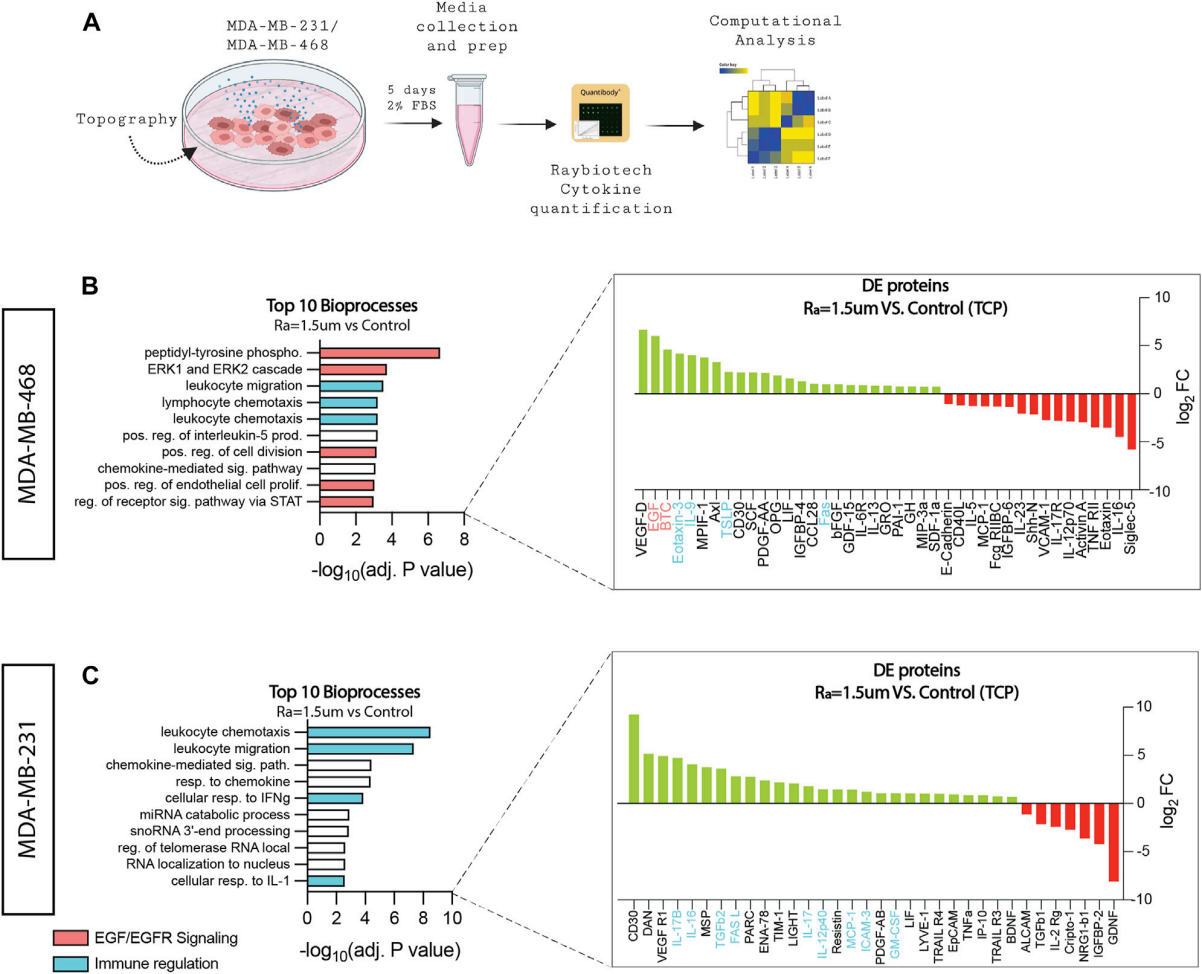
Surface roughness enhances bioprocesses associated with inflammation and EGF/EGFR signaling. **(A)** Schematic diagram of the workflow used to collect the conditioned media samples for cytokine quantification. **(B)** GO top 10 enriched bioprocesses and differentially upregulated (green) and downregulated (red) proteins of MDA-MB-468 cells cultured in surface roughness (Ra = 1.5 µm), revealing enhancement in pathways related to EGF/EGFR signaling and immune regulation (adj. *p* < 0.01). **(C)** GO top 10 enriched bioprocesses and differentially upregulated (green) and downregulated (red) proteins of MDA-MB-231 cells cultured in surface roughness (Ra = 1.5 µm), revealing enhancement in pathways related to immune regulation (adj. *p* < 0.01).

Increased tumor cell proliferation implies faster growth kinetics and, therefore, a more aggressive phenotype (Dai et al., 2005; Mester and Redeuilh, 2008). Such enhanced proliferative phenotypes are likely to be fulfilled by the secretion of endogenous cellular factors. Thus, the cytokine levels of cells were examined to identify proteins affected by surface topographies that can better inform how biological processes are affected at the cell level. A multiplex analysis of 200 human cytokines was performed on samples composed of both conditioned media and lysates derived from MDA-MB-231 and MDA-MB-468 cells cultured on surfaces with roughness Ra = 1.5 µm and compared to TCP control (Figure 2A). Gene ontology (GO) analysis identified that half of the enriched bioprocess in MDA-MB-468 cells cultured on surfaces with roughness Ra = 1.5 µm are related to EGF/EGFR signaling. Epidermal growth factor (EGF) and betacellulin (BTC), well-known ligands of the epidermal growth factor receptor (EGFR) family signaling pathway, were upregulated 64-folds and 23-folds, respectively (Figure 2B). It is common knowledge that the growth of MDA-MB-468 cells is driven by their EGFR-rich phenotype, suggesting that ligand overproduction induced by topographical stimuli could over-activate EGFR *via* autocrine signaling (Sigismund et al., 2018), thereby increasing cell proliferation. For MDA-MB-231, EGF/EGFR signaling was not enriched, which was expected because EGFR signaling in these cells is mostly associated with invasion and metastasis, a key characteristic phenotype of these cells (Price et al., 1999).

In addition to EGFR, one-third of the bioprocesses enriched in MDA-MB-468 were found to be related to immune regulation. Eotaxin-3 (CCL26), IL-9, TSLP, and Fas were differentially expressed (DE) on surfaces with Ra = 1.5 µm. These factors are responsible for the stimulation of immune cells to sites of inflammation (Goswami and Kaplan, 2011; Kawano et al., 2021) and immune cell death (Volpe et al., 2016), and cancer has been associated with immune privilege and increased invasiveness and metastasis (Abrahams et al., 2003; Lan et al., 2018; Das et al., 2021). Likewise, MDA-MB-231 cells cultured on surfaces with Ra = 1.5 µm showed enriched bioprocesses for inflammation (Figure 2C). Factors such as interleukins (IL-17, IL-12, IL-16, TGFβ, MCP-1, and GM-CSF have been associated with poor prognosis in breast cancer, attracting immune cells to the tumor and facilitating angiogenesis, inducing proliferation (Nam et al., 2008), metastasis (Dutta et al., 2018), and drug resistance (Zhang et al., 2022). Altogether, these results suggest that surface roughness stimuli amplifies TNBC oncogenic signals in TNBC.

**FIGURE 3:**
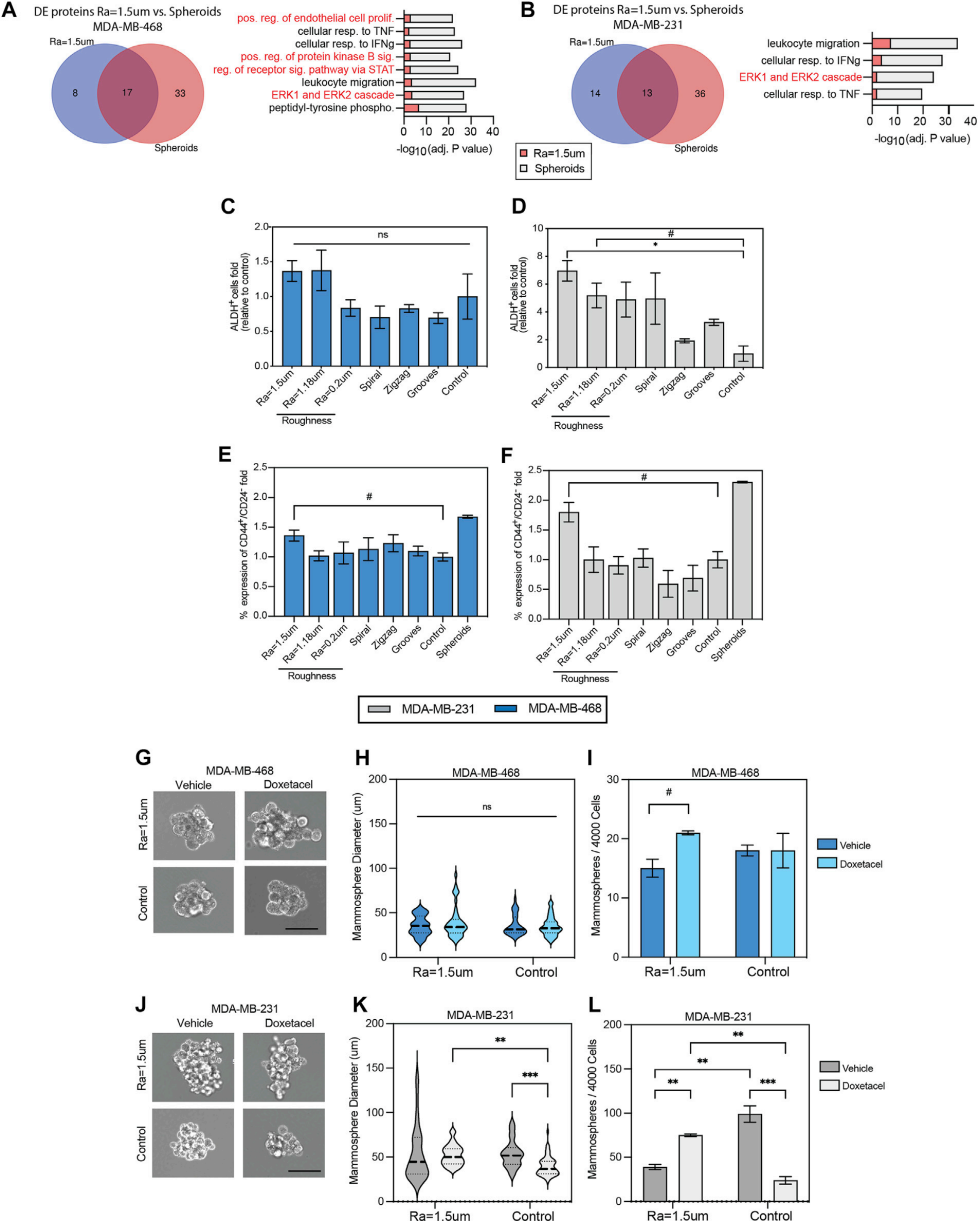
Surface roughness enhances the stemness of TNBC cells. Summary of differentially expressed proteins (fold change > 2 relative to TCP) in **(A)** MDA-MB-468 and **(B)** MDA-MB-231 cells cultured in surfaces with roughness Ra = 1.5 µm compared to 3D modality (spheroids) as a positive control for oncogenicity with barplot of GO enriched bioprocesses in common between both conditions and pathways related to stemness highlighted in red. Quantification of aldehyde dehydrogenase (ALDH) positive cells in **(C)** MDA-MB-468 and **(D)** MDA-MB-231 cell lines by flow cytometry, relative to TCP control. Quantification of CD44^+^/CD24^-^ subpopulations in **(E)** MDA-MB-468 and **(F)** MDA-MB-231 using flow cytometry, relative to TCP control. TNBC cells were cultured for 5 days in reduced serum (2% FBS) on the topographical substrates and TCP (control). **(G)** Representative images of MDA-MB-468 second generation mammospheres derived from cells cultured in rough surfaces and flat TCP. Cells were treated with Vehicle (DMSO) or Docetaxel (10 µM) for 7 days. Scale bar: 25 µm. **(H)** MDA-MB-468 second generation mammosphere’s size distribution. **(I)** Number of MDA-MB-468 second generation mammospheres formed (>50 µm) after 7 days of culture. **(J)** Representative images of MDA-MB-231 second generation mammospheres derived from cells cultured in rough surfaces and flat TCP. Cells were treated with Vehicle (DMSO) or Docetaxel (10 µM) for 7 days. Scale bar: 25 µm. **(K)** MDA-MB-231 second generation mammosphere’s size distribution. **(L)** Number of second generation MDA-MB-231 mammospheres formed (>50 µm) after 7 days of culture. Error bars depict the mean ± SEM of 2 independent experiments with n = 2-3 samples.*T*-test with Mann-Whitney non-parametric test. Asterisk (*) represent *p*-values < 0.05, (**) *p*-values < 0.01, (***) *p*-values < 0.001 and (****) *p*-values < 0.0001, (#) *p*-value<0.1.

Also, distinct factors related to cancer stemness are upregulated in cells cultured on rough surfaces. For example, MCP-1, TGFβ, and ICAM-3 were observed in MDA-MB-231 cells, whereas the EGFR ligands EGF and BTC were upregulated in MDA-MB-468 cells (Qadir et al., 2017; Shen et al., 2018; Lv et al., 2020; Viswanadhapalli et al., 2022). LIF and Fas were also upregulated in both cell lines. To further explore the effect of topographical stimuli on stemness, the enriched factor bioprocess profiles of TNBC cells cultured on surfaces with Ra = 1.5 µm were compared to 3D spheroids as a standard control of enriched cancer stem cells (CSC) in TNBC (Yilmazer, 2018). The factors and bioprocesses enriched in 3D spheroids were first identified relative to cell monolayers on TCP (Supplementary Figures S2A, B) prior to comparison with cells derived from rough surfaces. DE analysis revealed a total of 13 proteins shared between MDA-MB-231 cells cultured on roughness Ra = 1.5 µm and 3D spheroids. Accordingly, GO analysis identified four enriched bioprocesses in common, one of which was related to stemness (Figure 3A). Similarly, DE analysis of MDA-MB-468 cells cultured on roughness Ra = 1.5 µm and 3D spheroids identified 17 proteins in common with eight enriched mutual bioprocesses, of which 50% were linked to stemness (Figure 3B). To further confirm the enhanced CSC phenotype, the expression levels of the stemness markers, ALDH, CD44 and CD24 were examined in both TNBC cell lines. Overall, ALDH activity was increased on rough surfaces, but the levels varied across cell lines. A significant increase of almost 7-folds in the total fraction of ALDH + cells was observed in MDA-MB-231 cells cultured on surfaces with roughness Ra = 1.5 µm, relative to the TCP control (Figures 3C, D). This increase in ALDH + cells was correlated with a significant enrichment (∼70%) in the CD44^+^CD24^−^ CSC population. Similarly, the fraction of cells expressing ALDH+ and CD44^+^CD24-was enriched in MDA-MB-468 cultured on rough surfaces by 37.5% and 35.9%, respectively. The levels of CD44+/CD24-cell enrichment on rough surfaces were equivalent to those in tumor spheroids (Figures 3E, F), supporting relevant enrichment levels of the CSC phenotype (Ghuwalewala et al., 2016; Li et al., 2018). Overall, the CD44/CD24 cell distribution in TNBC cells was significantly altered across the surface topographies examined (Supplementary Figure S3), strengthening the importance of mechanical stimuli in modulating tumor stemness cell ratios. To further confirm that tumor stemness is a direct result from mechano stimulation, the analysis of ALDH levels per cell was stratified by regions within the geometrical micropatterns (curves and grooves). Although no significant differences were observed, a tendency of increased expression and of marginal significance for some instances was observed in areas of a higher mechanical stress/tension relative to flat areas such as the corners of the zig-zag patterns and the outer parts (edges) of the grooves. This observation is in agreement with prior studies that showed that geometric cues at perimeter features activate the expression of CSC markers of mouse melanoma cells (Lee et al., 2016), further reinforcing the impact of mechanical stress/tension altering the stemness phenotype in cultures (Supplementary Figures S4B, C).

To confirm the regenerative capacity and drug resistant phenotype of CSC enriched on rough surfaces, a single-cell suspension of unsorted cells was cultured in non-adherent plates to quantify the number of mammospheres formed and the sensitivity to docetaxel in second-generation mammospheres. The average size of MDA-MB-468 mammospheres derived from rough surfaces was comparable to that of the flat control (Figures 3G, H), however, the number of mammospheres derived from rough surfaces was higher in docetaxel-treated cultures than in the flat control (Figure 3I). Similarly, the average size of the MDA-MB-231 mammospheres derived from rough surfaces was significantly increased in docetaxel-treated cultures compared to the flat control (Figures 3J, K). Accordingly, the number of mammospheres also significantly increased in docetaxel-treated cultures derived from rough surfaces (Figure 3L). These results confirm the mammosphere-forming capacity and enriched chemoresistant phenotype of CSC derived from rough surfaces, which are well-known traits of CSCs in TNBC (Gómez-Miragaya et al., 2017; Xiong et al., 2020).

### 3.3 EGFR expression and drug efficacy is modulated in a roughness-dependent manner

**FIGURE 4:**
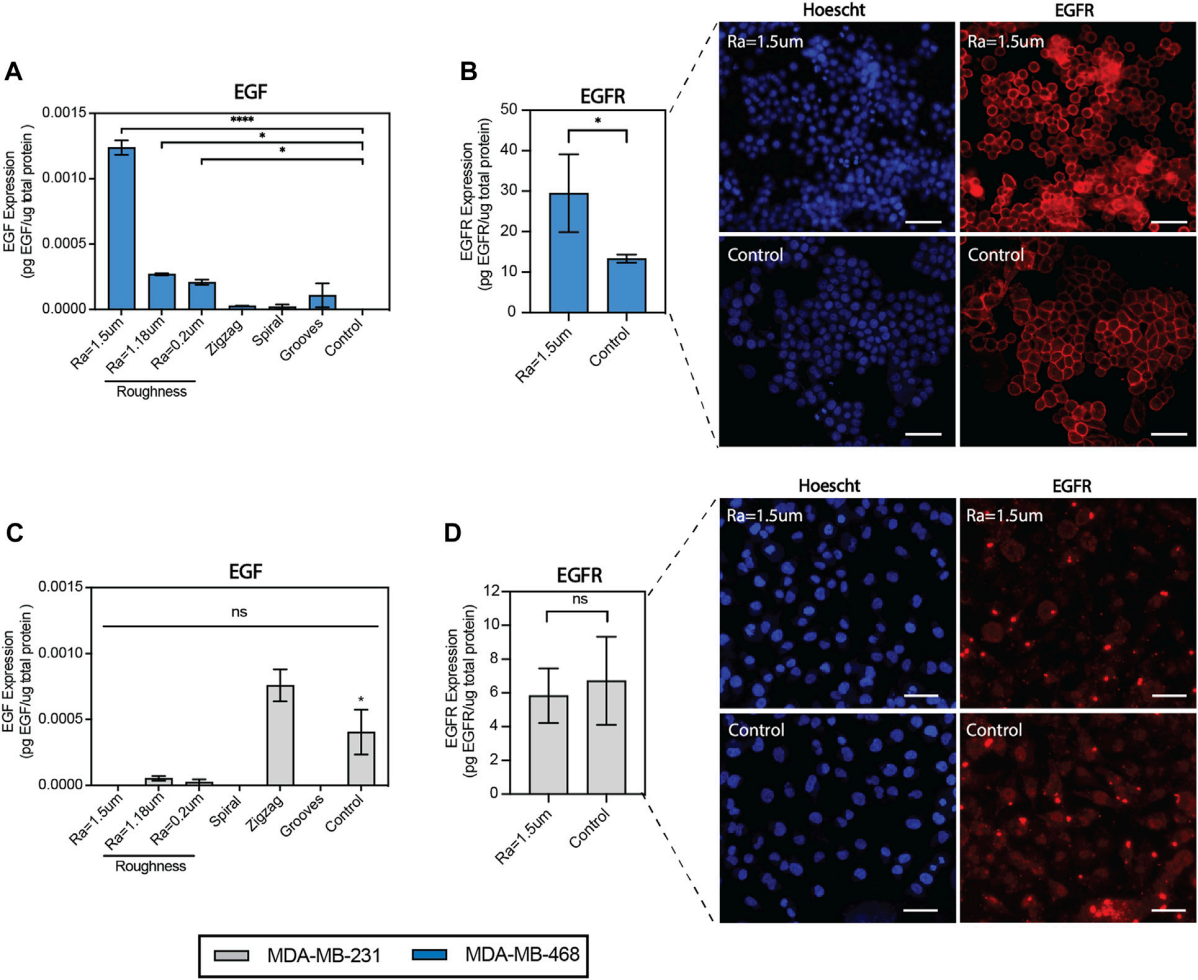
Surface roughness enhances EGF secretion and EGFR expression in MDA-MB-468 cells. **(A)** Quantification of epidermal growth factor (EGF) expression in MDA-MB-468 cultured on the topographical surfaces by enzyme-linked immunosorbent assays (ELISA). **(B)** Quantification of epidermal growth factor receptor (EGFR) expression in MDA-MB-468 cultured on surfaces with roughness Ra = 1.5 µm by enzyme-linked immunosorbent assays (ELISA), along with representative images of cells stained with anti-EGFR (red) and Hoechst (blue). Scale bar = 50 µm. **(C)** Quantification of epidermal growth factor (EGF) expression in MDA-MB-231 cultured on the topographical surfaces by enzyme-linked immunosorbent assays (ELISA). **(D)** Quantification of epidermal growth factor receptor (EGFR) expression in MDA-MB-231 cultured on surfaces with roughness Ra = 1.5 µm by enzyme-linked immunosorbent assays (ELISA), along with representative images of cells were stained with anti-EGFR (red) and Hoechst (blue). Scale bar = 50 µm. Error bars depict the mean ± SEM of 2 independent experiments with n = 2-3 samples. *T*-test with Mann-Whitney non-parametric test. Asterisk (*) represent *p*-values < 0.05, (**) *p*-values < 0.01, (***) *p*-values < 0.001 and (****) *p*-values < 0.0001.

To assess the impact of surface roughness on EGFR signaling, a quantitative analysis of EGFR/EGF activity was performed using enzyme-linked immunosorbent assay (ELISA). EGF secretion was confirmed to be significantly expressed in MDA-MB-468 cells cultured on surfaces with roughness Ra = 1.5 µm relative to the TCP control, as observed before in the proteome profiling (Figure 4A). In addition to EGF, EGFR levels were enhanced by almost 3-folds on rough surfaces relative to the TCP control (Figure 4B). EGFR was mainly distributed on the cell membrane of MDA-MB-468 cells, regardless of the topography, but was notably enhanced on rough surfaces. Flow cytometry confirmed EGFR enrichment on rough surfaces by enhancing EGFR expression levels per cell (Supplementary Figures S5A–C). EGFR + cells accounted for more than 97% of the total number of cells. We were not able to measure changes in the number of EGFR + cells. In bulk analysis of MDA-MB-231 cells, it was found that both EGF and EGFR levels were downregulated on rough surfaces, as both were either undetectable or low levels in the proteome analysis or ELISA assay, suggesting topographical suppression of EGF/EGFR signaling (Figures 4C, D). However, flow cytometry analysis showed a significant (2-fold) increase in the fraction of EGFR + cells on rough surfaces (Supplementary Figures S5A–C). A notable difference between MDA-MB-231 cells and MDA-MB-468 cells was the distribution of EGFR in the nucleus. Therefore, contrasting responses to topographical stimulation may be explained by differences in the proportion of EGFR + cells and EGFR localization within cells. Collectively, the data supports that surface roughness can upregulate both the EGFR expression levels and the number of positive cells, although in a cell line-dependent manner.

**FIGURE 5:**
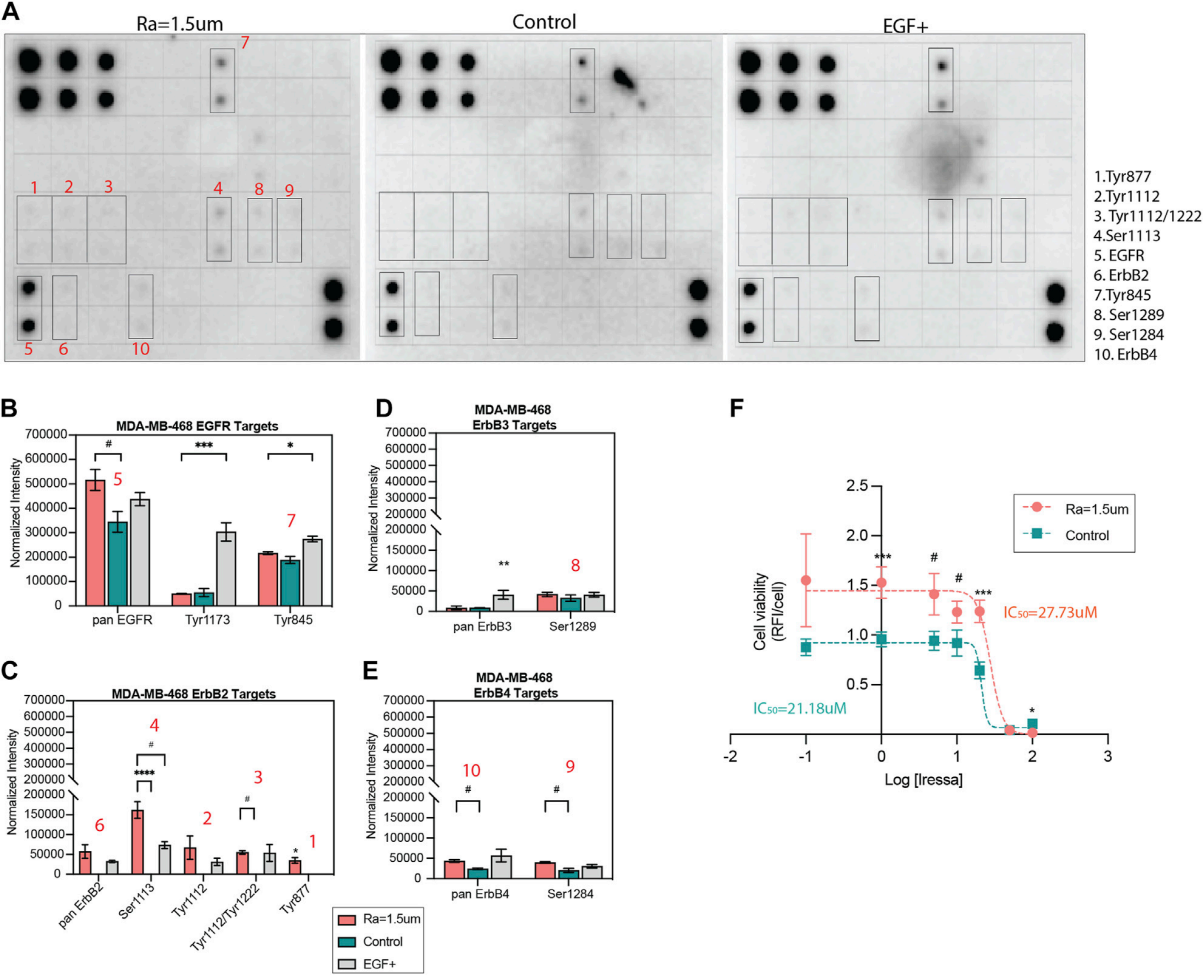
EGFR phosphorylation patterns and drug inhibition are altered by surface roughness in MDA-MB-468. **(A)** hEGFR phosphorylation micrographs of TNBC cells cultured for 5 days in reduced serum (2% FBS) on surfaces with roughness of Ra = 1.5 µm, TCP surface as a standard control or TCP surface supplemented with 10 ng/mL EGF as a positive control. The array includes 17 phospho-proteins of the pan EGFR, ErbB-2, ErbB-3, ErbB-4 receptors (identified in Supplementary Figure S6). Blots with increased expression are identified and numbered in red. **(B–E)** Quantification of the phosphorylation of pan EGFR, ErbB-2, ErbB-3, ErbB-4 and their corresponding phospho-proteins, depicted as the normalized intensity relative to assay positive controls. **(F)** EGFR inhibition-response curve of MDA-MB-468 cells cultured on surfaces with roughness Ra = 1.5 µm compared to TCP control. Error bars depict the mean ± SEM of 2-3 independent experiments with n = 2-3 samples. *T*-test with Mann-Whitney non-parametric test. Asterisk (*) represent *p*-values < 0.05, (**) *p*-values < 0.01, (***) *p*-values < 0.001 and (****) *p*-values < 0.0001, (#) *p*-values<0.1.

To further study the modulation of EGFR activity, the levels and patterns of 17 phosphorylation sites of the EGFR family of receptors were examined in TNBC cells. The phosphorylation levels of cells cultured on rough surfaces were compared to TCP (control) and exogenous supplementation of EGF ligand on TCP as a positive control for EGF/EGFR activity and quantified as the average intensity for sites that showed signals for at least one of these conditions. The results showed that the EGFR phosphorylation patterns changed with topographical stimuli of surface roughness and were cell line-dependent (Figure 5A). Consistent with our prior results, pan EGFR phosphorylation in MDA-MB-468 cells was significantly enhanced on the surface with roughness Ra = 1.5 µm relative to TCP (Figure 5B). The phosphorylation patterns across the four receptors and their residues were comparable between surfaces with roughness and EGF ligand (10 ng/mL) stimuli at most sites, with significant enhancements in Ser1113 and Tyr877 sites. Phosphorylation in pan ErbB2 (HER2) and various of its phosphorylation sites were only observed in response to topographical stimuli and exogenous EGF stimuli on TCP, while no signal was detected for the TCP control, suggesting a concentration-dependent effect (Figure 5C). Interestingly, phosphorylation at the Tyr877 site, associated with downstream processes related to migration in ovarian cancer and cell differentiation (Villa-Moruzzi, 2013; Broussard et al., 2021), was only detected in the rough surface condition. Similarly, surface roughness enhanced ErbB4 phosphorylation compared to the TCP control (Figures 5D, E) suggesting that factors other than EGF regulate EGFR activity in response to surface roughness. Enhanced EGF/EGFR signaling on surfaces with high roughness correlated with a reduction in drug sensitivity, as demonstrated by a significant increase in the IC50 dosage of gefitinib (Figure 5F), an EGFR tyrosine kinase inhibitor (Culy and Faulds, 2002). Such an altered drug response to Gefitinib in MDA-MB-468 and other TNBC models has been attributed to enhanced tumor stemness in prior studies (Nabholtz et al., 2014; Xu et al., 2018; Matossian et al., 2019), supporting the potential application of rough surface patterns to amplify the pool of heterogeneous tumor phenotypes in drug studies seeking to identify compounds with broader tumor efficacy (Hrustanovic et al., 2013; Bivona, 2020).

In MDA-MB-231 cells, the phosphorylation of EGFR family kinases was decreased on rough surfaces as compared to TCP (Supp. Figures 6A–F). Decreased EGFR phosphorylation relative to TCP was consistent with the decreased EGF ligand levels shown in Figure 4C. The phosphorylation site patterns of surface roughness were downregulated relative to those of the TCP control. The same phosphorylation sites that were affected in MDA-MB-468 cells were also affected in MDA-MB-231 cells; however, phosphorylation activity was consistently decreased in surfaces with roughness, contrary to what was observed in MDA-MB-468 cells. Surface roughness enhanced EGF/EGFR activity in MDA-MB-468 cells *via* autocrine signaling, whereas the opposite effect was observed in MDA-MB-231 cells, in which EGFR expression and activation were attenuated. This indicates that while mechanical stimuli on EGFR signaling are consistent across both cell types, the directionality of the cascade may be cell phenotype-dependent, where it is upregulated in non-invasive cells but downregulated in invasive cells.

**FIGURE 6:**
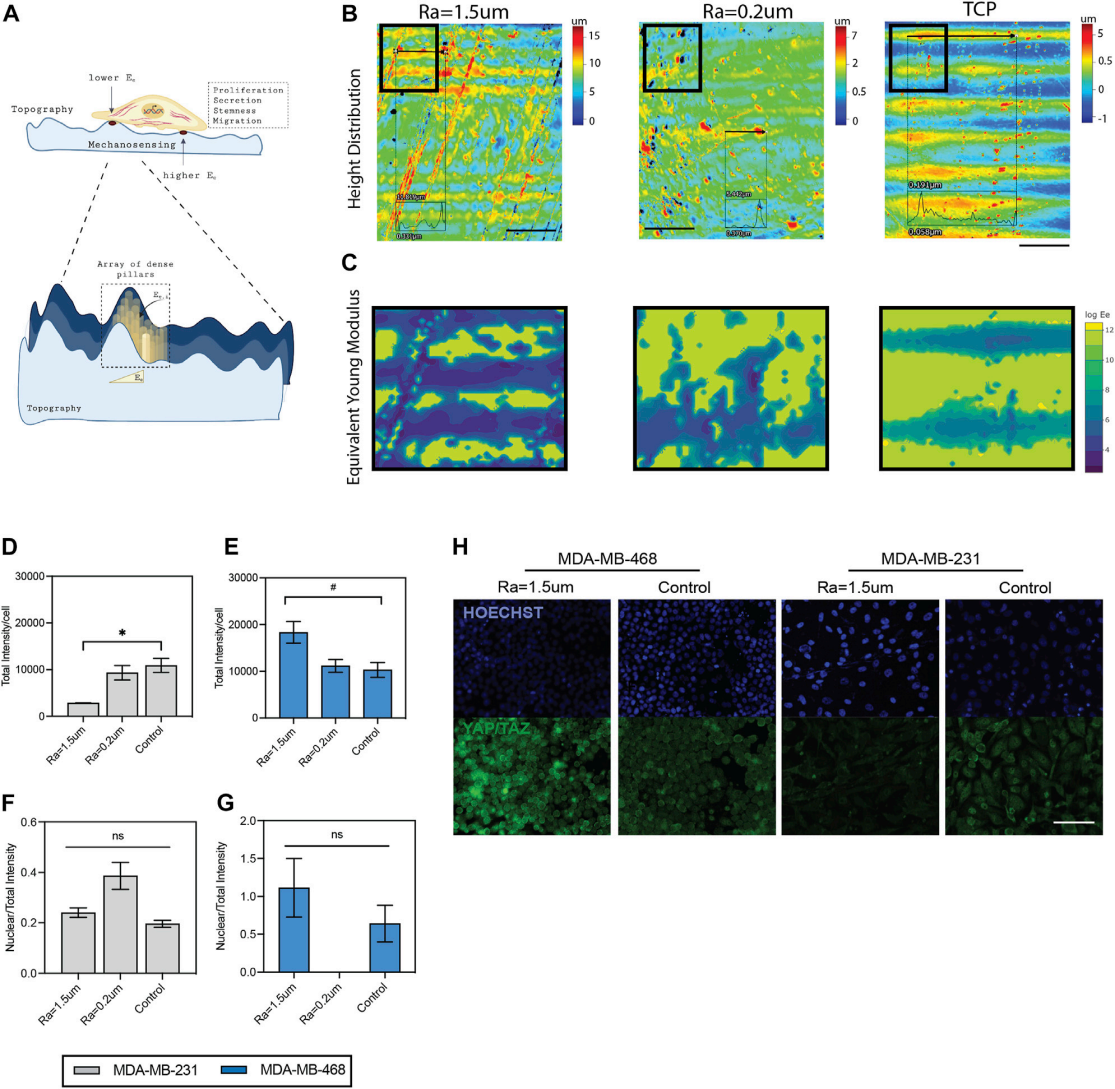
Mechanosensing of TNBC cells in surface roughness. **(A)** Schematic diagram of the mechanosensing of cells in rough surface. Surface forces were estimated as an array of dense pillars with variable heights, as described by (Wei, 2017). **(B)** Heatmap of height distribution of the surfaces. Scale bar: 500 µm. **(C)** Contour maps of estimated distribution of forces in the surfaces. Quantification of total YAP/TAZ per cell in **(D)** MDA-MB-231 and **(E)** MDA-MB-468 cells cultured in surfaces with roughness, compared to TCP control. Ratio between nuclear YAP/TAZ intensity and total YAP/TAZ intensity in **(F)** MDA-MB-231 and **(G)** MDA-MB-468 cells cultured in surfaces with roughness, compared to TCP control. **(H)** Representative immunofluorescence images of YAP/TAZ (green) and nuclei (blue) staining. Scale bar: 100 µm. Error bars depict the mean ± SEM of 2 independent experiments with n = 2 samples. *T*-test with Mann-Whitney non-parametric test. Asterisk (*) represent *p*-values < 0.05, (**) *p*-values < 0.01, (***) *p*-values < 0.001 and (****) *p*-values < 0.0001, (#) *p*-values<0.1.

Cell changes associated with surface roughness likely modulate cell behavior *via* a mechanosensing mechanism (Figure 6A). Microscale changes to the surface topography, such as the roughness of peak elevations, can modulate the effective modulus of elasticity (Ee) of the surface, where it has been demonstrated that surface height is inversely correlated with Ee (Wei, 2017). A summary of the estimated distribution of forces experienced by the cells in the roughness topographies compared to TCP is shown in Figure 6C, where surfaces with high roughness show increased areas of elevation in the surfaces (Figure 6B) compared to TCP, and thus a gradient of decreased forces in these areas of elevation (Figure 6C) (Wei, 2017). To further confirm that our topographies exerted mechano-sensing signals in the cells, the expression of two well-known mechanotransducers, Yes-associated protein (YAP) and the transcriptional coactivator with PDZ-binding motif (TAZ), was examined (Dupont et al., 2011; Cai et al., 2021). The results showed that the nuclear to total ratio of YAP/TAZ was not significantly affected by the surface roughness, but total YAP/TAZ levels were significantly altered for both cells (Figures 6D–H). This finding is in agreement with previous studies demonstrating an interplay between EGFR and YAP/TAZ in several cancers including TNBC, and their effect in tumorigenic properties (Cancer Discov, 2020; Ando et al., 2021; Moon et al., 2022; Soyama et al., 2022; Zhang and Li, 2022). Overall, this data confirms a mechanosensing effect in a roughness-dependent manner and suggests that changes in the EGF/EGFR signaling pathway are a result of altered YAP/TAZ levels driven by changes in surface roughness.

## 4 Discussion

Physical cues of the breast tumor microenvironment can influence cell behavior through an extent of changes in the mechanical properties of the extracellular matrix (ECM), such as stiffness and architecture, often linked to microstructure or topographies. Several studies using 2D cultures have demonstrated that stiffness regulates metastasis, CSC enrichments, and drug resistance of cancer cells (Wei et al., 2015; Hui et al., 2017; Tan et al., 2018). In the case of topographies, these are often neglected in cell-based studies primarily due to challenges in how these topographical patterns are translated to clinical models (Stallcop et al., 2018; Tudureanu et al., 2022). The lack or absence of studies that relate the biophysical cues to specific mechanisms and phenotypes has limited its rationale incorporation into cell-based assays. This study shows that topographical stimuli of the microenvironment regulate phenotypic changes of the bulk tumor cells. Major findings show that surface roughness influences the enrichment of CSCs and EGFR signaling activity in TNBC cells. We demonstrate that micropatterns generated by relatively simple methods can still exert significant changes in the behavior of TNBC cells, which implicates downstream influence in cell oncogenic activity, drug response and supporting their potential for cell-based assays.

Overexpression of EGFR is a well-known characteristic of TNBC cells and is related to poorer prognosis in patients (Park et al., 2014). In our study, we demonstrated that EGF ligand and receptor levels are modulated by surface roughness in TNBC cells, supporting that topographical sensing of cells in their microenvironment can guide them toward a more aggressive state. However, the directionality of the magnitude of the effect was cell-line dependent, highlighting the complexity of these cell-topographical interactions and the importance of the individual characterization of these stimuli. To our knowledge, this is the first report of EGFR modulation by topographical stimulation in TNBC cells. Nevertheless, previous studies have shown that matrix stiffness does regulate EGFR signaling and expression (Rosenthal, 2017; Saxena et al., 2017; Farahani et al., 2021). Also, the modulation of HER2 by topographical stimuli in HER2+ breast cancer cells was documented before by Daverey et al., where micro-gratings (grooves) were found to downregulate HER2 expression in HER2+ cell lines BT-474 and SKBr3 (Daverey et al., 2022). As prior findings, our results also confirmed that surface topography regulates HER2 phosphorylation. However, in our study, rough surfaces supported the re-expression of low levels of HER2 and phosphorylation levels in sites linked to EGFR-HER2 dimers in MDA-MB-468 cells. EGFR-HER2 dimerization produces a stronger activation of EGFR signaling, which translates into further hyperactivation of pathways associated with proliferation, angiogenesis, and metastasis (Jeon et al., 2017). While the expression of HER2 may be beneficial for HER2-targeted inhibitors, its re-expression in TNBC transfected cells implicates enhanced tumor growth and invasion, both hallmarks of aggressive tumor phenotypes (Li et al., 2020). In addition, our study showed that surface roughness modulates EGF/EGFR signaling and YAP/TAZ activity similarly. Our observation is consistent with prior results demonstrating EGFR-YAP/TAZ signaling interplay during tumor progression (Zanconato et al., 2016; Ando et al., 2021; Ortega et al., 2021; Vigneswaran et al., 2021; Zhang and Li, 2022), and further strengthens the fact that mechanical stimulation can drive changes in the EGF/EGFR signaling pathway impacting the oncogenic activity of TNBC. Thus, enrichments for EGFR-HER2 dimerization may be supported during therapy *via* mechanosensing signals triggered by changes in the matrix structure, further fulfilling drug resistance in TNBC. Yet, further studies are warranted to understand better the specific downstream biomolecular mechanisms driving these interactions.

In addition to EGFR activity, increased tumor stemness is one of the hallmarks of cancer and is a key feature of the progression and poor outcome. CSC are a population of stem-like cells with self-renewal and tumor-initiating capabilities and differentiation, shown to be responsible for tumor development, metastasis, and drug resistance (Chang, 2016). So far, many therapies target the fast-growing cells of the bulk tumor, yet, there are still challenges in understanding the mechanisms governing CSC and, thus, the development of CSCs-specific therapies (Franco et al., 2016; Zhou et al., 2021). Previous studies have demonstrated that surface topographies support the proliferation and maintenance of pluripotency markers of stem cells for optimal and scalable expansion (Reimer et al., 2016; Chaudhary and Rath, 2017). In the cancer field, previous studies have also demonstrated that surface nano-patterns and interfacial geometry enhance CD44+/CD24- ESA+ and other stem markers, highlighting the relevance of surface topographies to enrich CSC populations for isolation (Tan et al., 2015; Lee et al., 2016). Our study demonstrated the feasibility of surface roughness as a simple culture strategy to enrich CSC populations in TNBC cells in planar culture format. We observed differential upregulation of bioprocesses and cytokines associated with stemness, which matched with bioprocesses seen in 3D spheroid cultures. Also, these results correlated with enhanced CD44+/CD24-expression and ALDH + activity of the cells. One limitation of this study is the need for integrin-binding motifs on surfaces. We know that in tissues, CSC are subject to a combination of biochemical and mechanical stimuli, given mainly by integrin-matrix interactions. In contrast, in our 2D topographies, integrin-binding motifs are absent. Thus, future studies will seek to combine biochemical and physical stimuli to characterize the extent of cell phenotypes resulting from 2D topographies.

Although significant advances have been made in the development of technologies that allow a better understanding of the role of topographical features in cell behavior, a major limitation is their full availability to the scientific community. Microfabrication facilities involve high maintenance and instrumentation costs, technical expertise, and long prototyping times (Stallcop et al., 2018). Our study uses razor printing and surface polishing as straightforward microfabrication methods to generate topographical substrates on PS films. Contrary to classical microfabrication techniques (e.g., laser ablation, hot embossing, or lithography), our patterning methods have fast prototyping time, do not require technical expertise, have significantly lower equipment and material investment, and can be easily adapted into *in vitro* culture platforms for high-throughput analyses. One limitation is that our topographical array shows a higher heterogeneity in terms of the roughness levels generated and is restricted to the microscale level due to the inherent capabilities of our methods. Thus, emerging methods such as thermal shrinking may produce more micro-scale reproducibility (Sayed and Selvaganapathy, 2022).

## 5 Conclusion

This study demonstrates that surface topographies can modulate the oncogenic activity of TNBC cells. The proteome of TNBC cells cultured on rough surfaces was enriched for cytokines associated with metastasis, proliferation, immune regulation, and stemness. Moreover, enhanced phenotypic markers of CSC populations were confirmed in planar cultures on rough surfaces. The EGF/EGFR activity was found as a novel target of mechanical stimuli driven by surface roughness. Overall, this work demonstrates the potential of surface roughness patterns as a viable culture strategy to enrich CSC populations in drug studies seeking to identify compounds with broader efficacy.

## Data Availability

The raw data supporting the conclusion of this article will be made available by the authors, without undue reservation.

## Supplementary material

SUPPLEMENTARY FIGURE S1 Morphology of TNBC cells cultured in topographical substrates. Quantification of cytoskeleton **(A,B)** area and **(C, D)** elongation of MDA-MB-231 and MDA-MB-468 cell lines cultured for 5 days in reduced serum (2% FBS) on the topographical substrates relative to the control (TCP). Error bars depict the mean ± SEM of 3 independent experiments with *n* = 3-4 samples. *T*-test with Mann-Whitney non-parametric test. Asterisk (*) represent *p*-values < 0.05, (**) *p*-values < 0.01, (***) *p*-values < 0.001 and (****) *p*-values < 0.0001.

SUPPLEMENTARY FIGURE S2 Enriched bioprocesses in TNBC 3D spheroid cultures. **(A)** GO top 10 enriched bioprocesses and differentially upregulated (green) and downregulated (red) proteins of MDA-MB-468 3D spheroids (adj. *p* <0.01). **(B)** GO top 10 enriched bioprocesses and differentially upregulated (green) and downregulated (red) proteins of MDA-MB-231 spheroids (adj. *p* <0.01). Proteins highlighted in red are associated with cancer stemness.

SUPPLEMENTARY FIGURE S3 Expression of stem markers in TNBC cultured on topographical substrates. **(A)** Representative images of CD44+ (FITC) cells flow cytometry intensity histograms for Ra=1.5 µm and TCP compared to isotype control. Distribution of CD44/CD24 subpopulations in **(B)** MDA-MB-231 and **(C)** MDA-MB-468 cultured in the topographical substrates. **(D)** Representative flow cytometry plots of the distribution of the ALDH1+ subpopulation for Ra=1.5 µm and TCP surfaces gated to ALDH1 inhibitor control.

SUPPLEMENTARY FIGURE S4 Geometric features of the topographical substrates affect TNBC cells phenotypes. **(A)** Single cell analysis of EdU+ cells in distinct regions of the spiral, zigzag and groove micropatterns. **(B)** Quantification of the stemness marker ALDH1 in distinct regions of the spiral, zigzag and groove micropatterns. A total of 35 to 40 cells were analyzed per region. **(C)** Representative images of ALDH1 expression of MDA-MB-231 cells located in the corners and linear regions of the zigzag micro patterns. Cells were stained with anti-ALDH1 (red) and hoechst (blue). Scale bar = 100 µm. Error bars depict the mean ± SD of 1 independent experiment with *n* = 2-3 samples.*T*-test with Mann-Whitney non-parametric test. Asterisk (*) represent *p*-values < 0.05, (**) *p*-values < 0.01, (***) *p*-values < 0.001, (****) *p*-values < 0.0001 and (#) *p*-values < 0.10.

SUPPLEMENTARY FIGURE S5 EGFR expression in TNBC cells. **(A)** Representative flow cytometry plots of the distribution of the EGFR+ population of cells cultured on Ra = 1.5 µm and TCP surfaces gated to the isotype control. Flow cytometry analysis of **(B)** The percentage of EGFR+ cells and **(C)** The expression of EGFR in cells cultured on surfaces with roughness Ra = 1.5 µm and TCP (control), depicted by the mean fluorescent intensity (MFI) per cell. Error bars depict the mean ± SEM with N = 3. Statistical significance was determined by a T-test. Asterisk (*) represent *p*-values < 0.05, (**) *p*-values < 0.01, (***) *p*-values < 0.001, (****) *p*-values < 0.0001 and (#) p-values < 0.10.

SUPPLEMENTARY FIGURE S6 EGFR phosphorylation patterns are altered in MDA-MB-231 cells cultured on rough surfaces. **(A)** Blot analysis and ID’s of the phosphorylation array. **(B)** hEGFR phosphorylation micrographs of TNBC cells cultured for 5 days in reduced serum (2% FBS) on surfaces with roughness of Ra = 1.5 µm, TCP surface as a standard control or TCP surface supplemented with 10 ng/mL EGF as a positive control. The array includes 17 phospho-proteins of the pan EGFR, ErbB-2, ErbB-3, ErbB-4 receptors (identified in part A). Blots with increased expression are identified and numbered in red. **(B–F)** Quantification of the phosphorylation of pan EGFR, ErbB-2, ErbB-3, ErbB-4 and their corresponding phospho-proteins, depicted as the normalized intensity relative to assay positive controls. Error bars depict the mean ± SEM of 2 independent experiments with *n* = 2 samples.*T*-test with Mann-Whitney non-parametric test. Asterisk (*) represent *p*-values < 0.05, (**) *p*-values < 0.01, (***) *p*-values < 0.001 and (****) *p*-values < 0.0001, (#) *p*-values<0.1.

## Glossary

ALDH: Aldehyde dehydrogenase 1
BSA: bovine serum albumin
BTC: betacellulin
CSC: cancer stem cells
DE: differentially expressed
DMEM: dulbecco’s modified eagle’s medium
ECM: extracellular matrix
EGF: epidermal growth factor
ELISA: enzyme-linked immunosorbent assay
ER: estrogen receptor
ESA: epithelial specific antigen
FBS: fetal bovine serum
GM-CSF: Granulocyte-macrophage colony-stimulating factor
GO: gene ontology
HER1/EGFR: epidermal growth factor receptor
HER2/ErbB2: epidermal growth factor receptor 2
HER3/ErbB3: epidermal growth factor receptor 3
HER4/ErbB4: epidermal growth factor receptor 4
ICAM-3: Intercellular adhesion molecule 3
IL: interleukin
LIF: leukemia inhibitory factor
MCP-1: monocyte chemoattractant protein 1
PBS: phosphate buffer saline
PS: polystyrene
PR: progesterone receptor
Ra: average roughness
SEM: standard error of the mean
TCP: tissue culture plastic
TGFb1: transforming growth factor beta 1
TME: tumor microenvironment
TNBC: triple-negative breast cancer
TSLP: thymic stromal lymphopoietin
