# Supplementary figures and images for: Surface roughness modulates EGFR signaling and stemness of triple-negative breast cancer cells
Source: Front Cell Dev Biol. 2023 Mar 8;11:1124250. doi: 10.3389/fcell.2023.1124250 (PMC10030610; doi:10.3389/fcell.2023.1124250)

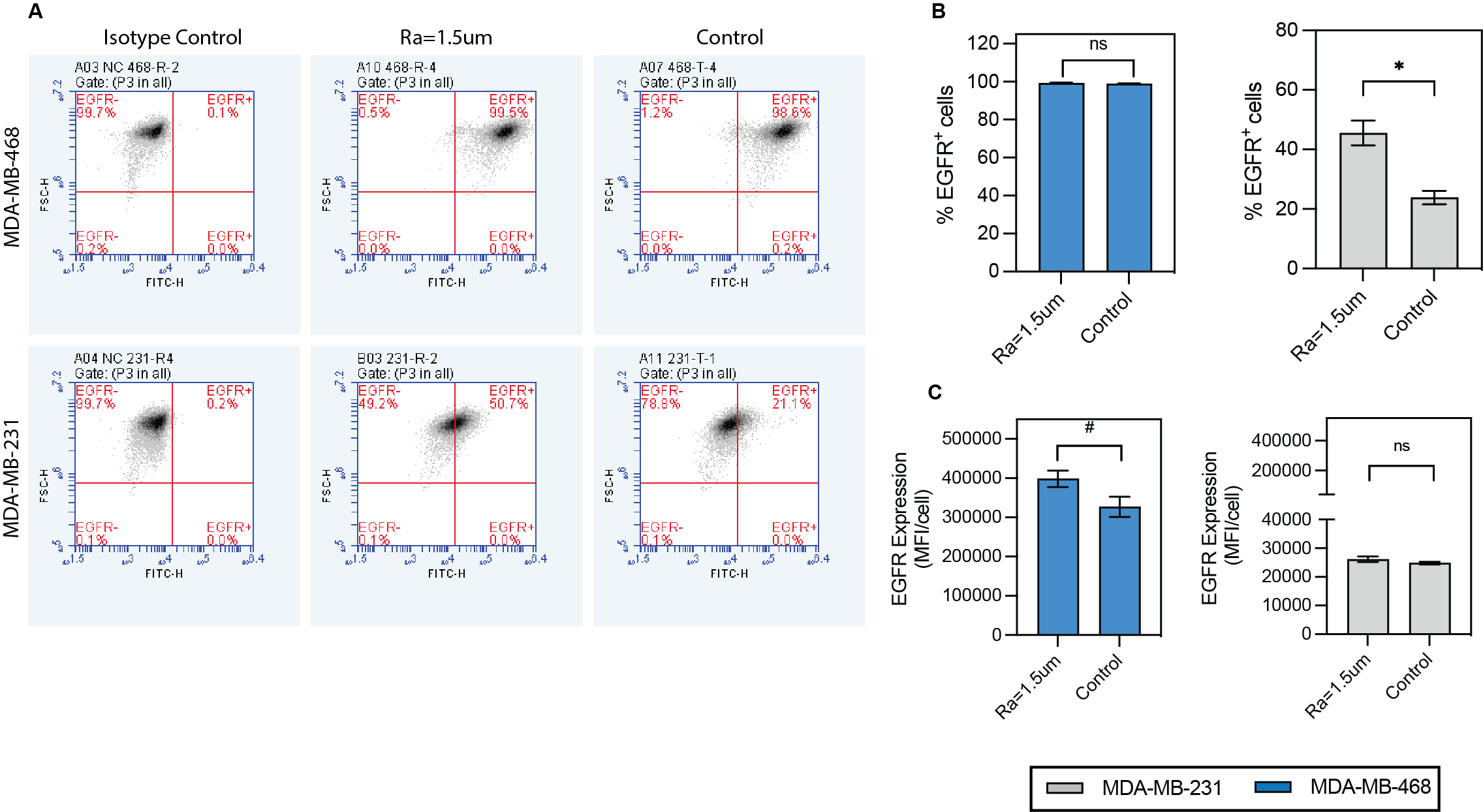

Supplement: Supplementary file 1 [file Image5.PNG]

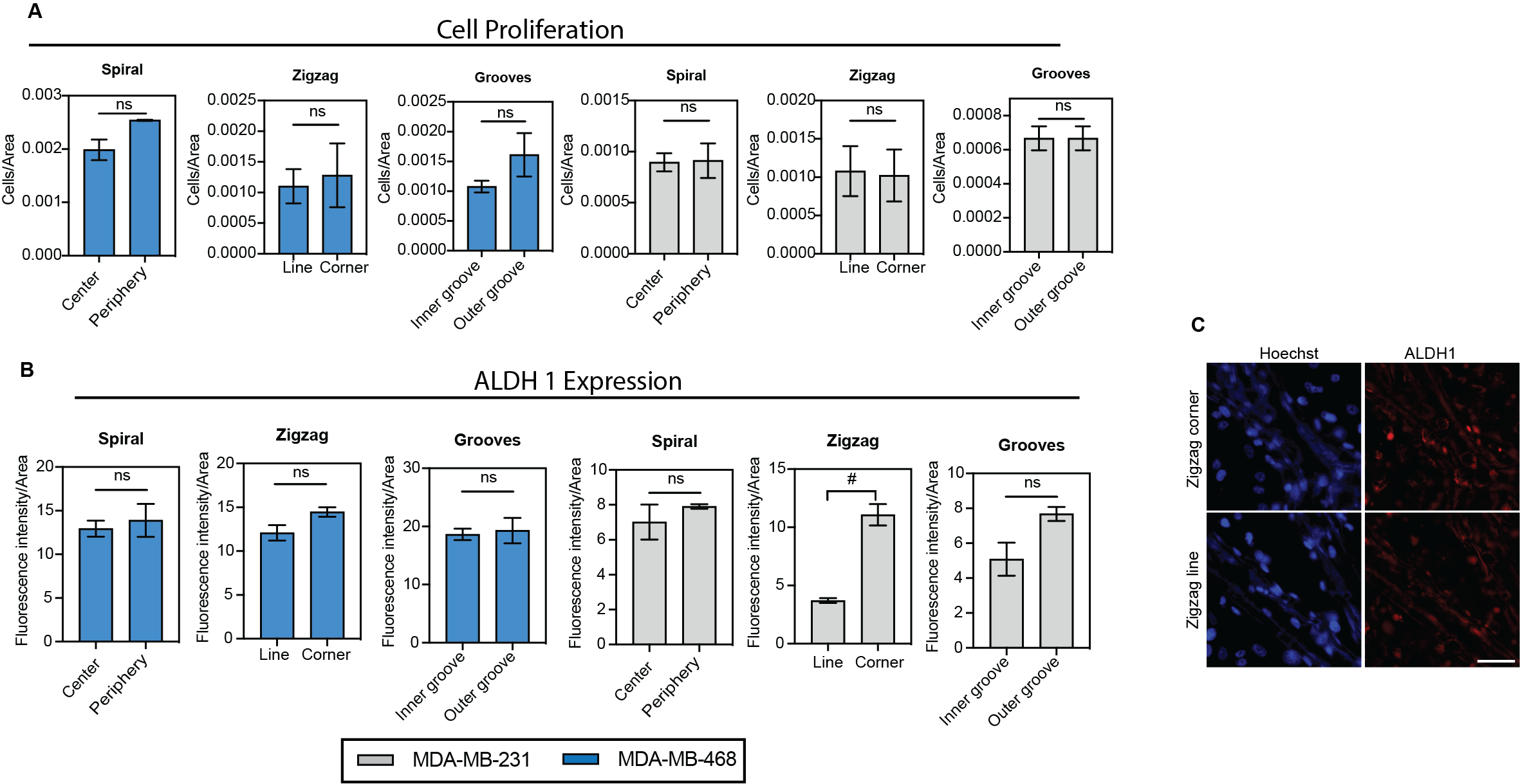

Supplement: Supplementary file 2 [file Image4.PNG]

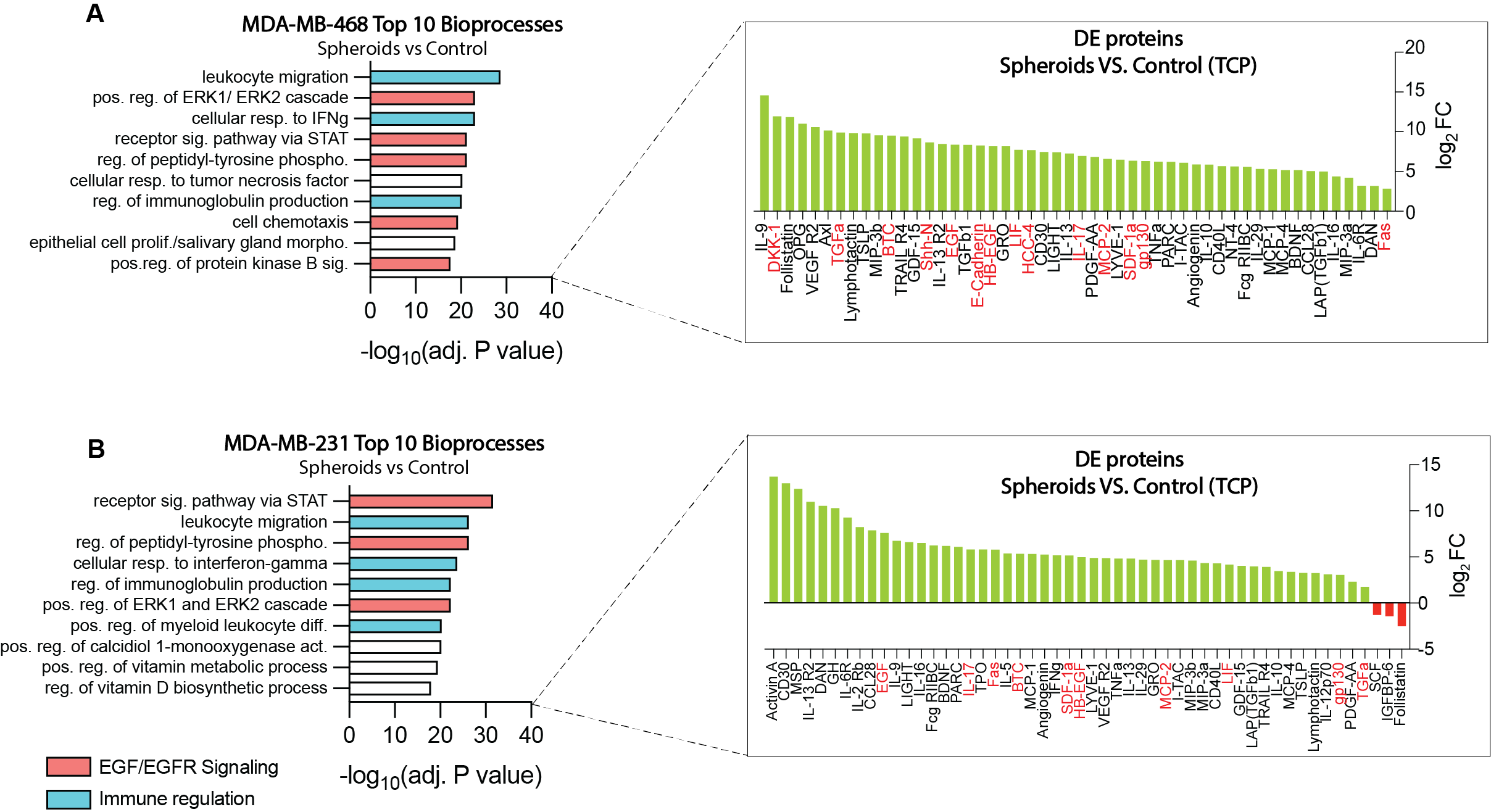

Supplement: Supplementary file 3 [file Image2.PNG]

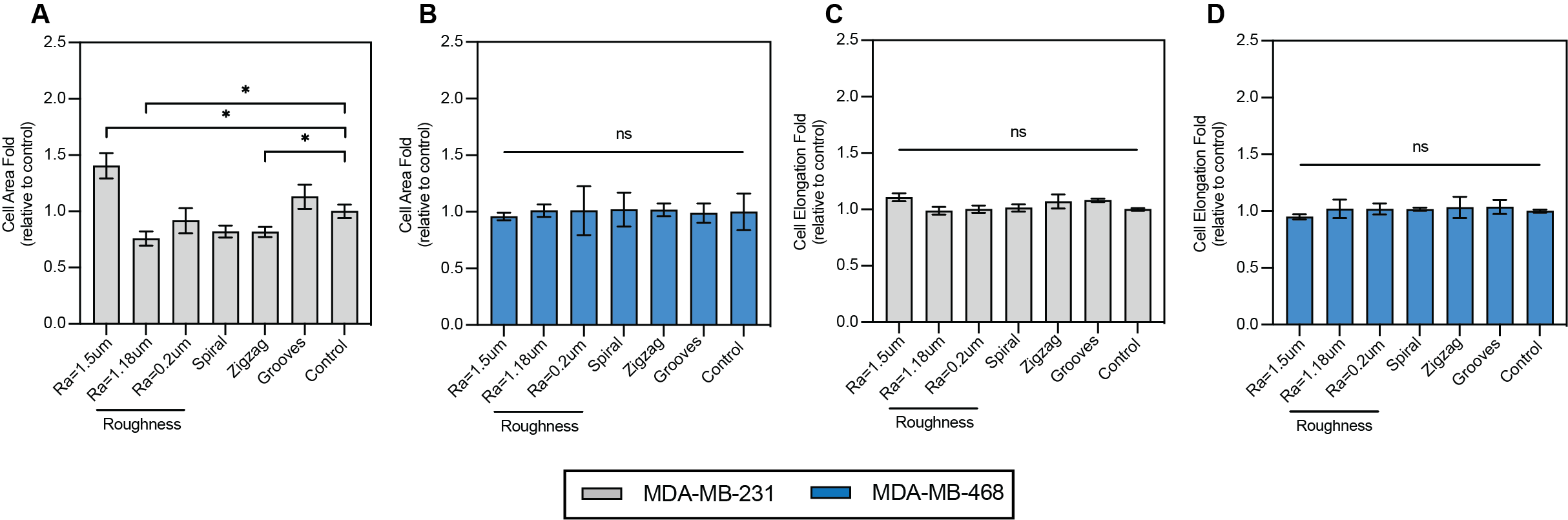

Supplement: Supplementary file 4 [file Image1.PNG]

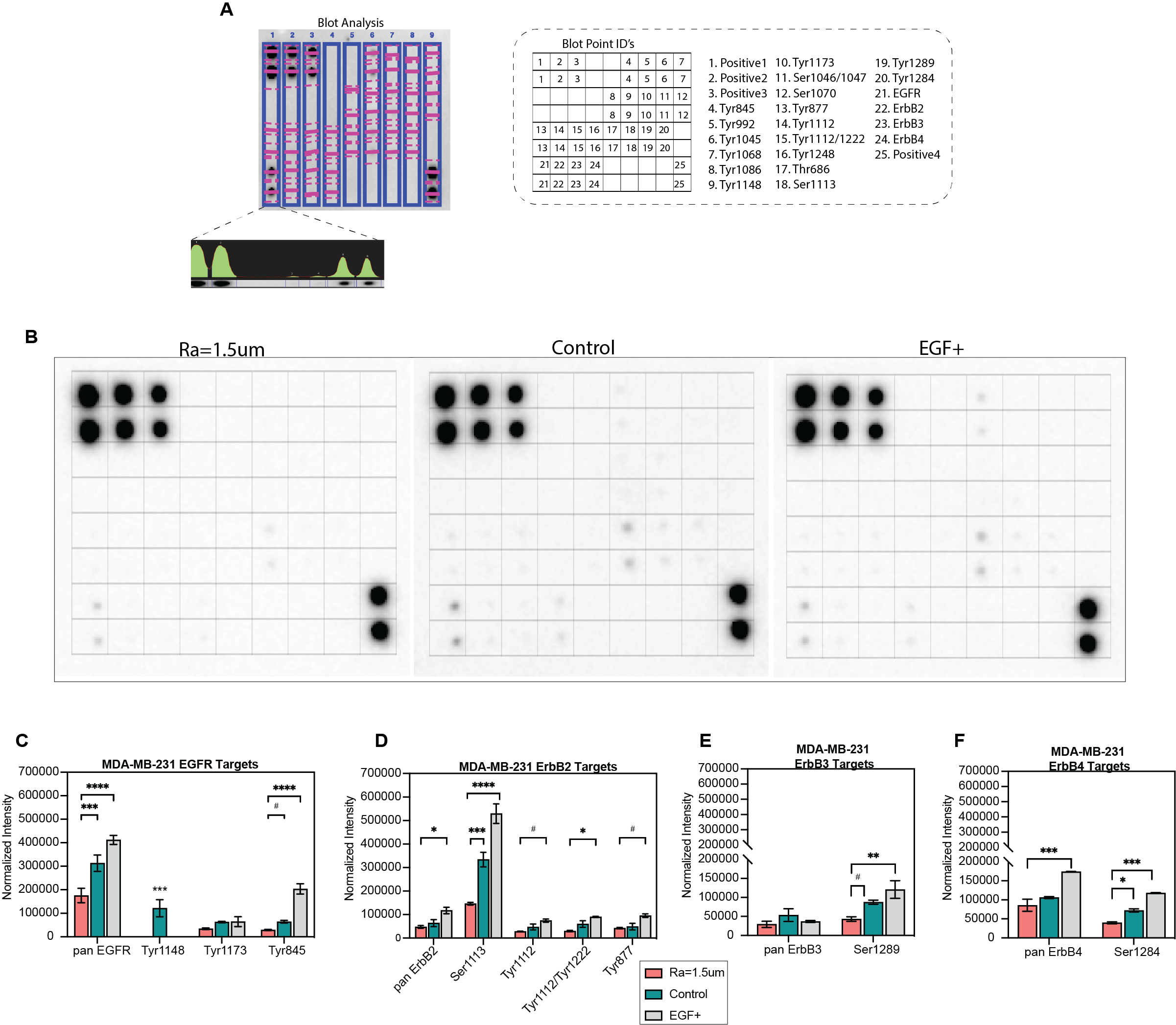

Supplement: Supplementary file 5 [file Image6.PNG]

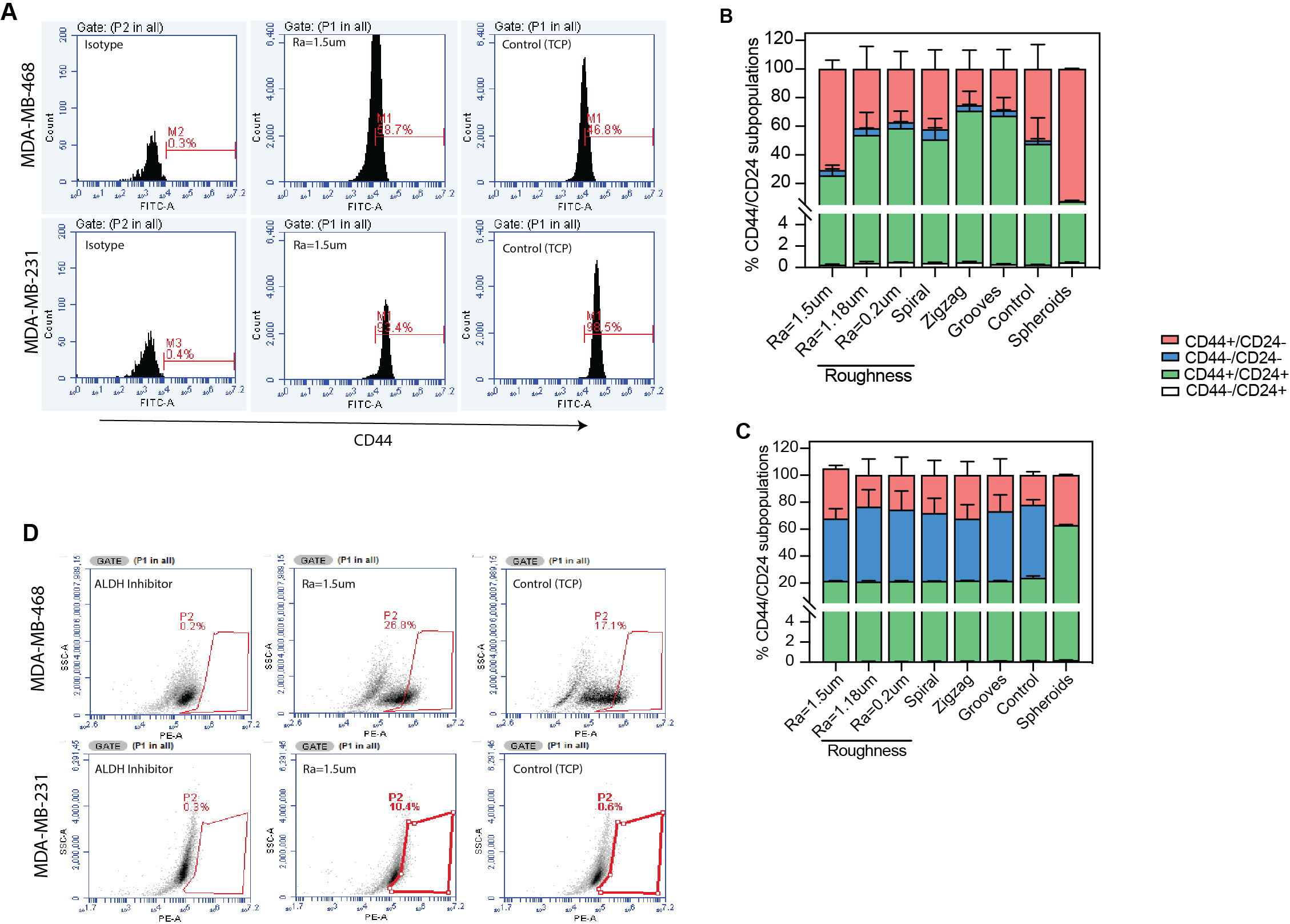

Supplement: Supplementary file 6 [file Image3.PNG]
